# Supplementary material for: Basal Mitophagy Occurs Independently of PINK1 in Mouse Tissues of High Metabolic Demand
Source: Cell Metab. 2018 Feb 6;27(2):439–449.e5. doi: 10.1016/j.cmet.2017.12.008 (PMC5807059; doi:10.1016/j.cmet.2017.12.008)
Supplement: Document S1. Figures S1–S4 [file mmc1.pdf]

Cell Metabolism, Volume 27

Supplemental Information

Basal Mitophagy Occurs Independently of PINK1

in Mouse Tissues of High Metabolic Demand

Thomas G. McWilliams, Alan R. Prescott, Lambert Montava-Garriga, Graeme Ball, François Singh, Erica Barini, Miratul M.K. Muqit, Simon P. Brooks, and Ian G. Ganley

Basal mitophagy occurs independently of PINK1 in mouse tissues of high-metabolic demand

Thomas G. McWilliams, Alan R. Prescott, Lambert Montava-Garriga, Graeme Ball, François Singh, Erica Barini, Miratul M. K. Muqit, Simon P. Brooks and Ian G. Ganley

Supplemental Figures

Figure S1: Monitoring mitophagy/autophagy using *mito*-QC/mCherry-GFP-LC3 and mass spectrometry sequence coverage. Related to Figure 1.

(A) DFP triggers mitophagy in *Pink1* WT and KO *mito*-QC MEFs. MEFs were treated with DMSO (Control) and DFP for 24 hours. Scale bar, 5 μm. (B) Quantitation of mitophagy induced by iron-chelation in S1A (Two-way ANOVA, ****=P<0.0001). (C) Endogenous activation of the PINK1-Parkin pathway. Adult fibroblasts were established from *Pink1* WT and KO mice and stimulated ±CCCP for 18 hours. Enriched mitochondrial fractions were processed for ubiquitin capture by $TUBE^{UBA2}$ as previously described. Denatured protein extracts were subjected to SDS-PAGE and immunoblotting for the Parkin substrate, CISD1. Membranes were stripped and re-probed with antibodies to the PINK1 substrate, phospho-S65 Ubiquitin. (D) Targeting strategy for mCherry-GFP-LC3 mice used in the study, generated by Taconic Artemis GmbH. (E) Mouse PINK1 sequence coverage from WT cortex and cerebellum. Unique peptides highlighted in red exhibited a Mascot ion score >22 (indicating identity or extensive homology).

Figure S2: *mito*-QC reveals mitophagy in the dopaminergic system *in vivo.* Related to Figure 2.

(A) Immunoblot showing no differences in levels of GFP, and the OMM Protein Tom20 in total brain extracts from *Pink1* WT and KO *mito*-QC mice. (B) Isosurface volume render of midbrain dopaminergic neurons with high levels of mitochondrial turnover in cell bodies. Inset shows a representative image from the original raw stack used to generate the isosurface render. (C) Additional quantative parameters measured using *mito*-QC. No differences were observed between genotypes in the

mean size or shape of mitolysosomes in midbrain dopaminergic axons or OB PGNs (n.s.=*P*>0.05; *Student's t*-test). (D) Mitophagy in A16 DA periglomerular neurons of the olfactory bulb (OB); G denotes a glomerulus. Arrows indicate mitophagy within PGNs (E) Analyses of mitophagy in all PGN DA neurons from *Pink1* WT and KO animals reveal no differences between genotypes (Student's *t*-test; n.s.=*P*>0.05). All scale bars, 5 µm. (F) *mito*-QC illuminates the mitochondrial rich nature of olfactory glomeruli. Airysan imaging and isosurface rendering revealed an unexpected complex and dense mitochondrial meshwork in adult olfactory glomeruli, and the intimate associations with TH-positive A16 PGNs. (G) Maximum projection z-stack showing representative example of mitophagy in a GFAP-positive astrocyte *in vivo*. Scale bar, 5 *µ*m.

Figure S3: *mito*-QC reveals mitophagy in highly metabolic tissues. Related to Figures 3 and 4.

(A) Section of adult retina with associated extra-ocular muscle. Mitophagy is visible in the ONL, and in muscle shown in transverse and longitudinal orientations. (B) Mitophagy proceeds independently of the visual cycle. Representative images of ONL showing no differences in retinal mitophagy in animals during light or dark cycles. (C) Additional quantitative parameters of mitolysosomes and mitochondrial cell biology: no differences were observed (n.s.=*P*>0.05; *Student's t*-test), apart from a modest decrease in mitolysosome size in KO microglia (*=*P*<0.05; *Student's t*-test). (D) Airyscan image of adult pancreas from a *mito*-QC animal, showing distinct pools of mitochondria previously reported by Petersen and colleagues. PG-M: perigranular mitochondria; PN-M: perinuclear mitochondria; SP-M: subplasmalemmal mitochondria.

Figure S4: Loss of PINK1 does not influence 3-NPA mitotoxicity *in vivo*. Related to Figure 4.

(A) Analysis of mitophagy reveals no differences in the striata, striatal vasculature and hearts of *Pink1* WT and KO mice treated systemically with the brain-penetrant mitotoxin 3-NPA for 7 days (n.s.=*P*>0.05; One-Way ANOVA with *Bonferroni post-hoc* test). (B) 3-NPA does not activate the endogenous PINK1-Parkin pathway. IP shows stabilization of endogenous PINK1 protein with CCCP, but not 3-NPA treatment. (C) Composite tile-scan micrograph showing a transverse section of hindlimb skeletal

muscle. The highly oxidative fibers of the soleus (S) and highly glycolytic fibers of the white gastrocnemius (GC) are easily distinguished by mitochondrial content using GFP expression of *mito*-QC. Dashed lines delineate different anatomical boundaries of the GC-white (Gw), GC-mixed (Gm) and GC-red (Gr) zones. In this anatomical orientation, *mito*-QC reveals the lateral-medial gradient of glycolytic to oxidative muscle fibers. Shown are representative examples of GC/S muscles treated with 3-NPA from *Pink1* WT and KO *mito*-QC mice. Close-up images reveal the striking detail of the mitochondrial reticulum in different sub-regions. Scale bars = 500, 200 and 10 µm, respectively. (D) Quantitation of mitochondrial content as a function of GFP intensity. A non-significant, yet observable elevation is evident in the skeletal muscles of *Pink1* KO animals treated with 3-NPA. (E) Quantitation of mitophagy as a function of increasing mitochondrial content in 3-NPA treated *Pink1* WT and KO mice (*=P<0.05; 3-NPA treated *Pink1* WT vs. KO using One-Way ANOVA, and *Bonferroni*'s post-hoc test to compare all conditions within regions).

Figure S1

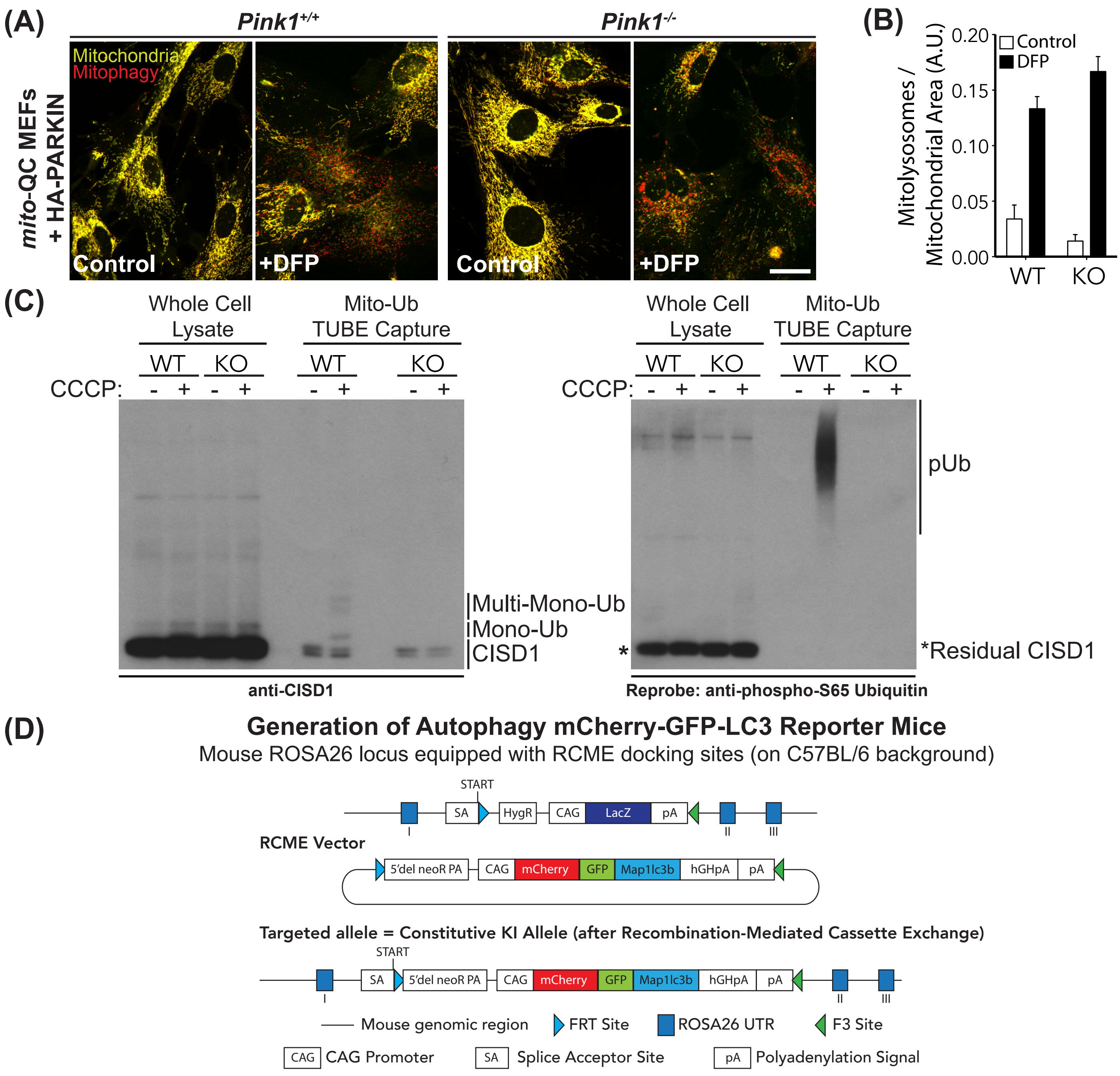

(E)

PINK1 peptides identified from PINK1 IP by mass spectrometry

Cortex (CTX) WT - 21% Sequence Coverage	Cerebellum (CB) WT - 18% Sequence Coverage
1 MAVRQALGRG LQLGRALLLR FAPKPGPLFG WGK**PGPAAAW** **GRGERPGQVV**	1 MAVRQALGRG LQLGRALLLR FAPK**PGPLFG** **WGKPGPAAAW** **GRGERPGQVV**
51 **SPGAQPR**PVG LPLPDRYRFF R**QSVAGLAAR** IQRQFMVRAR GGAGPCGRAV	51 **SPGAQPR**PVG LPLPDRYRFF R**QSVAGLAAR** IQRQFMVRAR GGAGPCGRAV
101 FLAFGLGLGL IEEKQAEGRR **AASACQEIQA** **IFTQK**TKRVS DPLDTRCWQG	101 FLAFGLGLGL IEEKQAEGRR **AASACQEIQA** **IFTQK**TKRVS DPLDTRCWQG
151 FR**LEDYLIGQ** **AIGK**GCNAAV YEATMPTLPQ HLEKAKHLGL IGKGPDVVLK	151 FR**LEDYLIGQ** **AIGK**GCNAAV YEATMPTLPQ HLEKAKHLGL IGKGPDVVLK
201 **GADGEQAPGT** **PTFPFAIK**MM WNISAGSSSE AILSK**MSQEL** **VPASRVALAG**	201 **GADGEQAPGT** **PTFPFAIK**MM WNISAGSSSE AILSK**MSQEL** **VPASR**VALAG
251 **EYGAVTYR**RS RDGPKQLAPH PNIIRVFRAF TSSVPLLPGA LADYPDMLPP	251 EYGAVTYRRS RDGPKQLAPH PNIIRVFRAF TSSVPLLPGA LADYPDMLPP
301 HYYPEGLGHG RTLFLVMKNY PCTLR**QYLEE** **QTPSSR**LATM MTLQLLEGVD	301 HYYPEGLGHG RTLFLVMKNY PCTLR**QYLEE** **QTPSSR**LATM MTLQLLEGVD
351 HLVQQGIAHR DLKSDNILVE WDSDGCPWLV ISDFGCCLAD QHVGLR**LPFN**	351 HLVQQGIAHR DLKSDNILVE WDSDGCPWLV ISDFGCCLAD QHVGLRLPFN
401 **SSSVER**GGNG SLMAPEVSTA HSGPSAVIDY SKADTWAVGA IAYEIFGLAN	401 SSSVERGGNG SLMAPEVSTA HSGPSAVIDY SKADTWAVGA IAYEIFGLAN
451 PFYGQGSAHL ESRSYQEAQL PEMPESVPPE ARRLVRSLLQ REASKRPSAR	451 PFYGQGSAHL ESRSYQEAQL PEMPESVPPE ARRLVRSLLQ REASKRPSAR
501 LAANVLHLSL WGEHLLALKN LKLDKMIAWL LQQSAATLLA DRLREKSCVE	501 LAANVLHLSL WGEHLLALKN LKLDKMIAWL LQQSAATLLA DRLREKSCVE
551 TKLQMLFLAN LECEALCQAA LLLSSWRAAP	551 TKLQMLFLAN LECEALCQAA LLLSSWRAAP

Figure S2

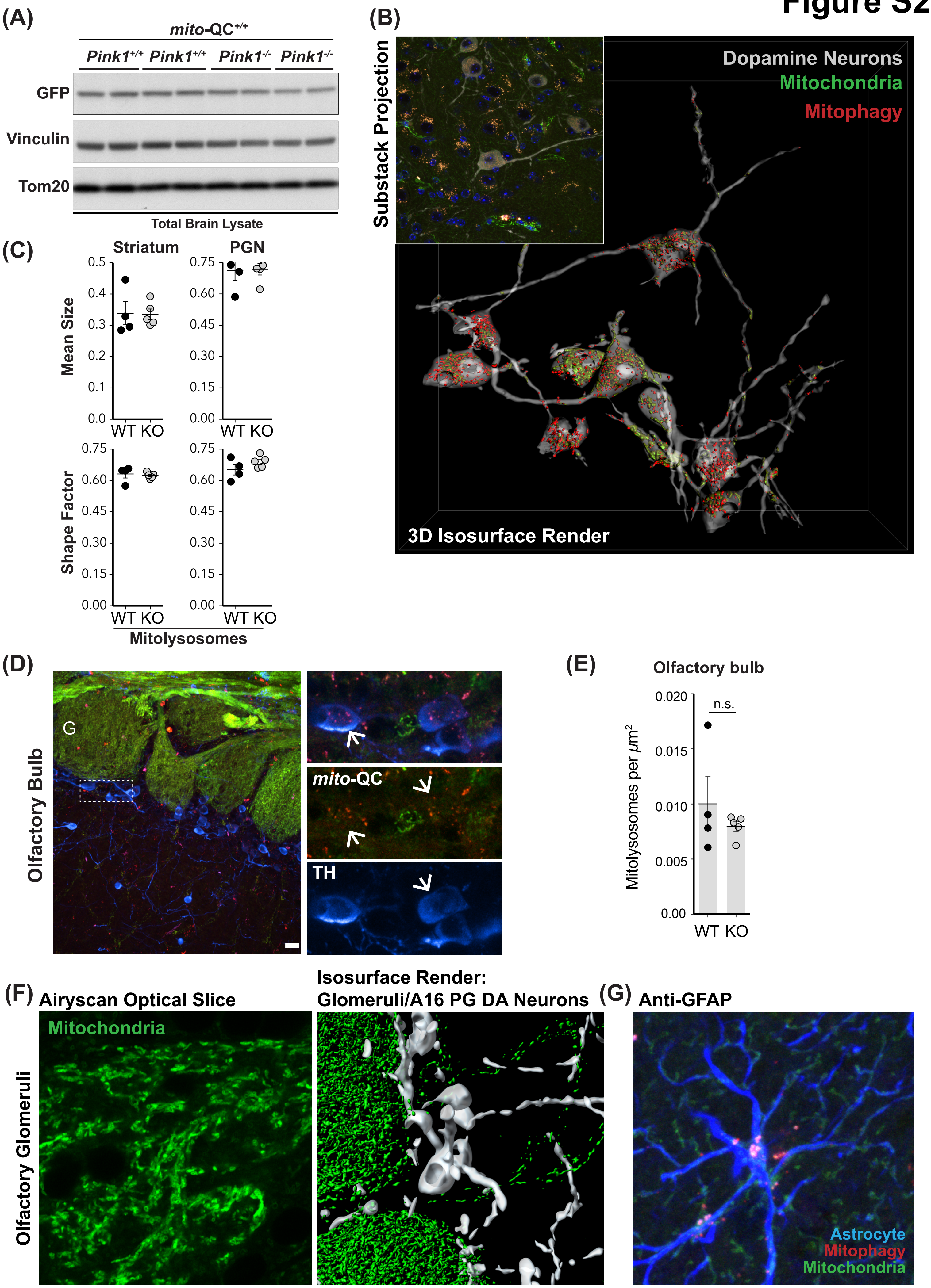

(A)
mito-QC+/+
Pink1+/+ Pink1+/+ Pink1-/- Pink1-/-
GFP
Vinculin
Tom20
Total Brain Lysate
(B)
Substack Projection
Dopamine Neurons
Mitochondria
Mitophagy
3D Isosurface Render
(C)
Striatum
PGN
Mean Size
Shape Factor
WT KO
Mitolysosomes
(D)
Olfactory Bulb
G
mito-QC
TH
(E)
Olfactory bulb
n.s.
Mitolysosomes per μm²
WT KO
(F)
Airyscan Optical Slice
Mitochondria
Olfactory Glomeruli
Isosurface Render:
Glomeruli/A16 PG DA Neurons
(G)
Anti-GFAP
Astrocyte
Mitophagy
Mitochondria

Figure S3

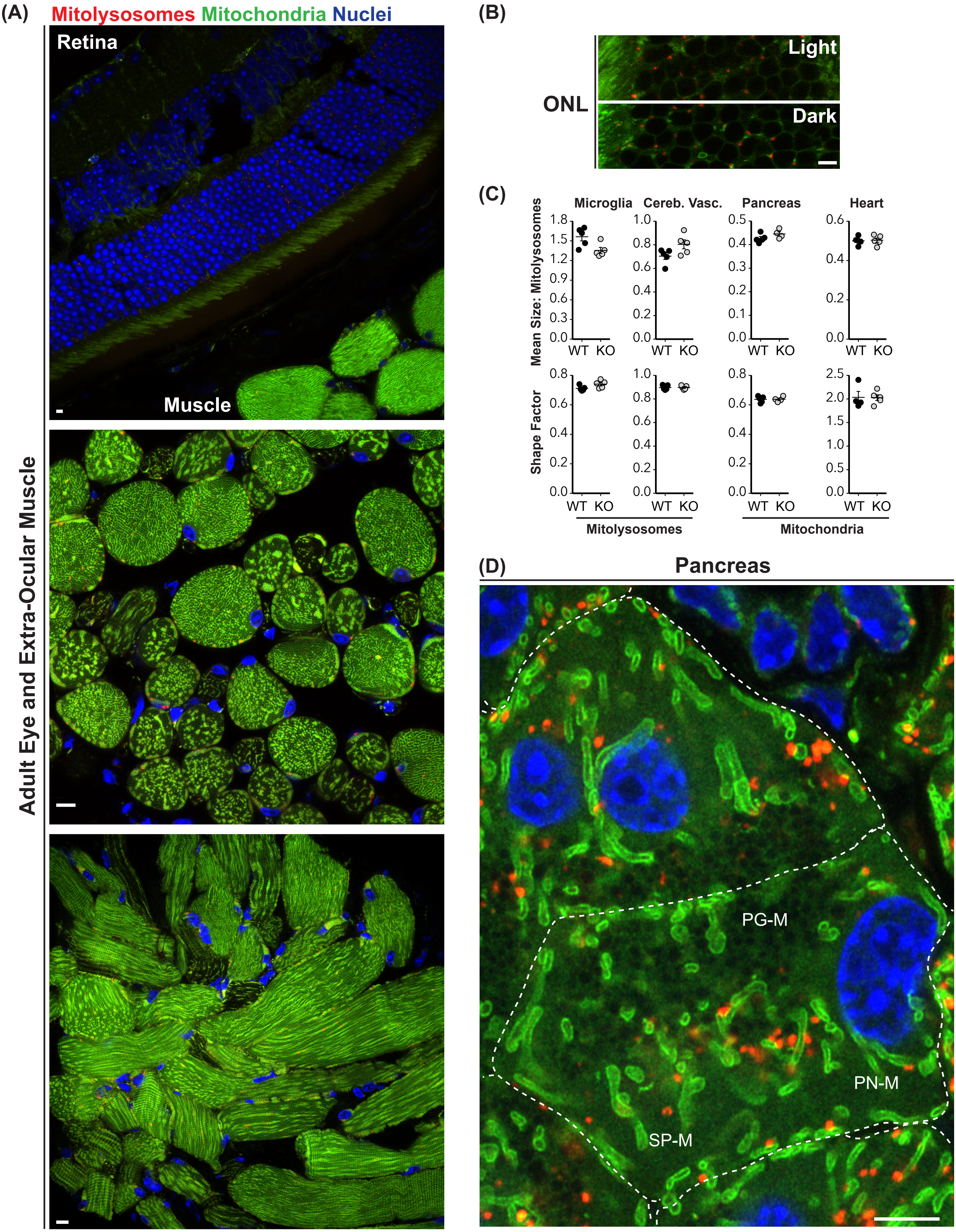

(A)
Mitolysosomes Mitochondria Nuclei
Retina
Muscle
Adult Eye and Extra-Ocular Muscle
(B)
ONL
Light
Dark
(C)
Mean Size: Mitolysosomes
Shape Factor
Microglia
Cereb. Vasc.
Pancreas
Heart
WT KO
Mitolysosomes
Mitochondria
(D)
Pancreas
PG-M
PN-M
SP-M

Figure S4

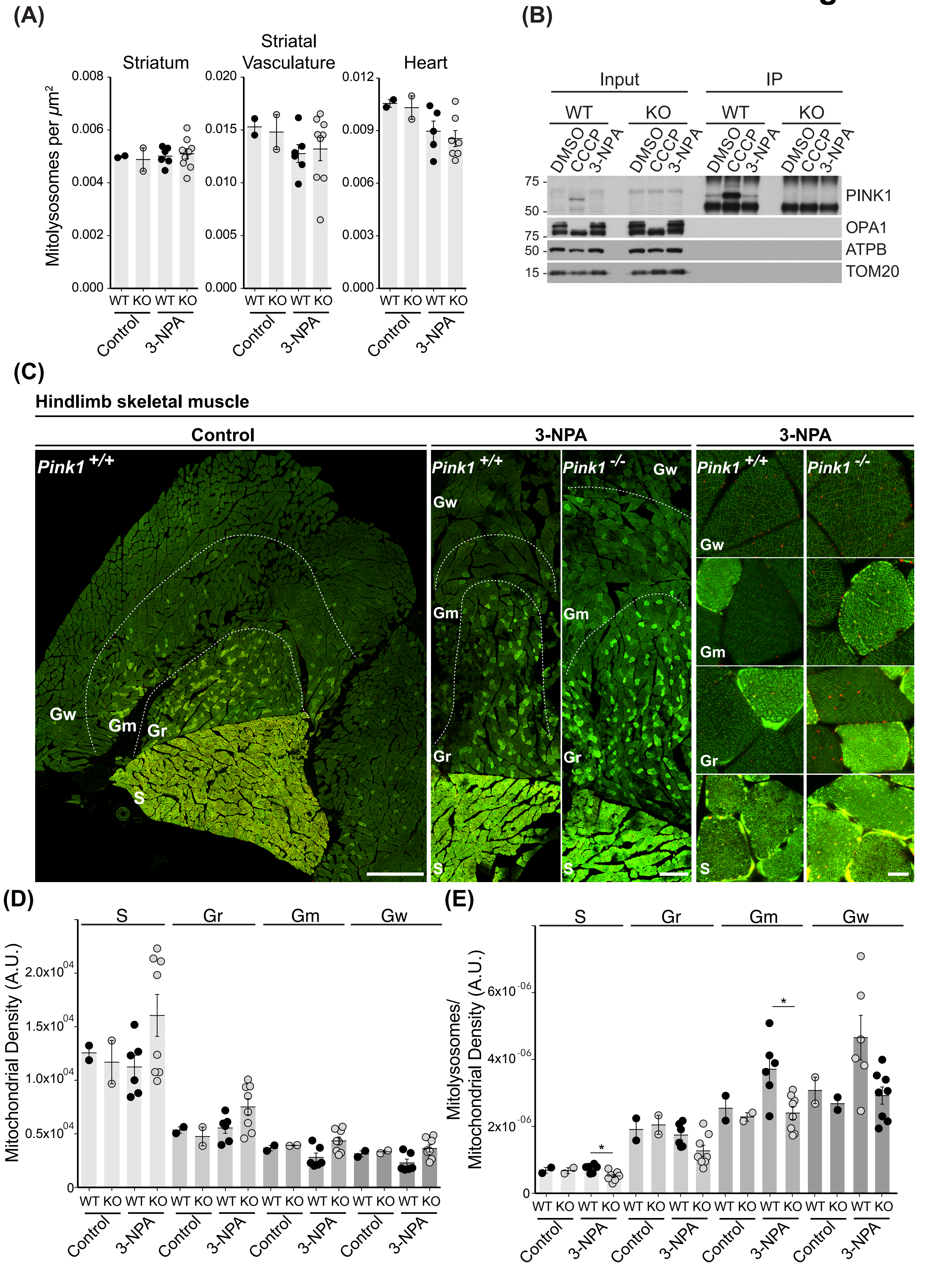
(A)
Striatum
Striatal Vasculature
Heart
Mitolysosomes per μm²
0.008
0.006
0.004
0.002
0.000
0.020
0.015
0.010
0.005
0.000
0.012
0.009
0.006
0.003
0.000
WT KO WT KO
Control
3-NPA
(B)
Input
IP
WT
KO
DMSO CCCP 3-NPA
75
50
75
50
15
PINK1
OPA1
ATPB
TOM20
(C)
Hindlimb skeletal muscle
Control
3-NPA
Pink1 +/+
Pink1 -/-
Gw
Gm
Gr
S
(D)
S
Gr
Gm
Gw
Mitochondrial Density (A.U.)
2.0x10^04
1.5x10^04
1.0x10^04
0.5x10^04
0
(E)
Mitolysosomes/ Mitochondrial Density (A.U.)
6x10^-06
4x10^-06
2x10^-06
0
*
