# Supplementary material for: Basal Mitophagy Occurs Independently of PINK1 in Mouse Tissues of High Metabolic Demand
Source: Cell Metab. 2018 Feb 6;27(2):439–449.e5. doi: 10.1016/j.cmet.2017.12.008 (PMC5807059; doi:10.1016/j.cmet.2017.12.008)
Supplement: Document S2. Article plus Supplemental Information [file mmc2.pdf]

Short Article

Cell Metabolism

# Basal Mitophagy Occurs Independently of PINK1 in Mouse Tissues of High Metabolic Demand

## Graphical Abstract

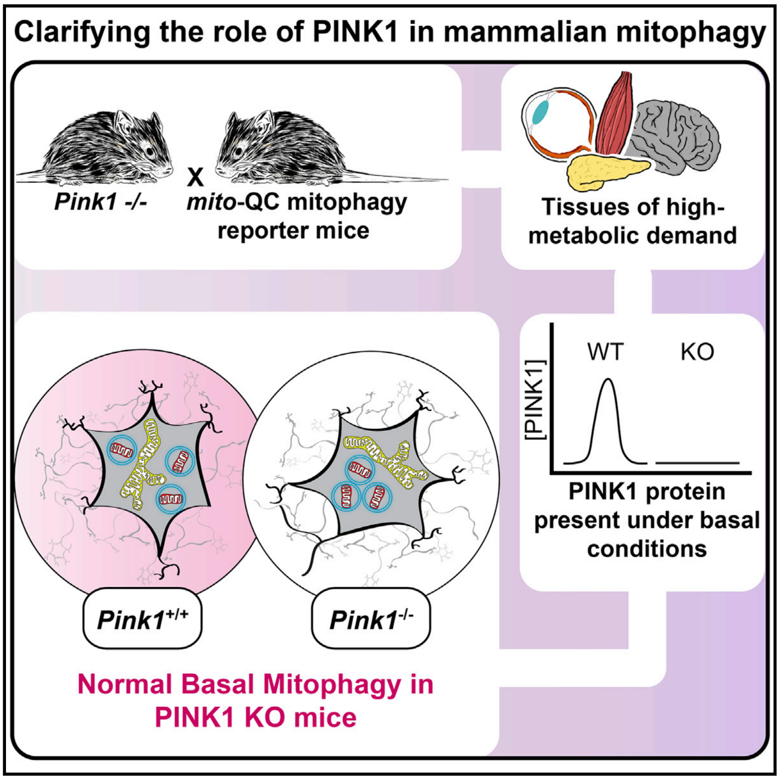


## Highlights

- Endogenous PINK1 protein is detectable in mouse tissues
- PINK1 is differentially expressed in both neural and non-neural tissues
- Highly metabolic tissues exhibit differential levels of basal mitophagy
- Basal mitophagy is comparable between PINK1 wild-type and knockout mice

## Authors

Thomas G. McWilliams, Alan R. Prescott, Lambert Montava-Garriga, ..., Miratul M.K. Muqit, Simon P. Brooks, Ian G. Ganley

## Correspondence

thomas.mcwilliams@helsinki.fi (T.G.M.), i.ganley@dundee.ac.uk (I.G.G.)

## In Brief

The Parkinson's disease protein PINK1 is regarded as a master regulator of mitophagy. McWilliams et al. discover that endogenous PINK1 is dispensable for basal mitophagy *in vivo*. Furthermore, they define a rigorous framework to detect endogenous PINK1 protein in mouse tissues. This reveals the complex, context-dependent nature of mammalian mitophagy.

McWilliams et al., 2018, Cell Metabolism *27*, 439–449
February 6, 2018 © 2017 The Author(s). Published by Elsevier Inc.
https://doi.org/10.1016/j.cmet.2017.12.008

Cell Metabolism
Short Article

# Basal Mitophagy Occurs Independently of PINK1 in Mouse Tissues of High Metabolic Demand

Thomas G. McWilliams,[1,*] Alan R. Prescott,[2] Lambert Montava-Garriga,[1] Graeme Ball,[2] François Singh,[1] Erica Barini,[1] Miratul M.K. Muqit,[1,3] Simon P. Brooks,[4] and Ian G. Ganley[1,5,*]
[1]MRC Protein Phosphorylation and Ubiquitylation Unit
[2]Dundee Imaging Facility, School of Life Sciences
University of Dundee, Dundee DD1 5EH, UK
[3]School of Medicine, University of Dundee, Dundee, DD1 9SY, UK
[4]The Brain Repair Group, Division of Neuroscience, School of Biosciences, Cardiff University, Cardiff CF10 3AX, UK
[5]Lead Contact
*Correspondence: thomas.mcwilliams@helsinki.fi (T.G.M.), i.ganley@dundee.ac.uk (I.G.G.)
https://doi.org/10.1016/j.cmet.2017.12.008

## SUMMARY

**Dysregulated mitophagy has been linked to Parkinson's disease (PD) due to the role of PTEN-induced kinase 1 (PINK1) in mediating depolarization-induced mitophagy *in vitro*. Elegant mouse reporters have revealed the pervasive nature of basal mitophagy *in vivo*, yet the role of PINK1 and tissue metabolic context remains unknown. Using *mito*-QC, we investigated the contribution of PINK1 to mitophagy in metabolically active tissues. We observed a high degree of mitophagy in neural cells, including PD-relevant mesencephalic dopaminergic neurons and microglia. In all tissues apart from pancreatic islets, loss of *Pink1* did not influence basal mitophagy, despite disrupting depolarization-induced Parkin activation. Our findings provide the first *in vivo* evidence that PINK1 is detectable at basal levels and that basal mammalian mitophagy occurs independently of PINK1. This suggests multiple, yet-to-be-discovered pathways orchestrating mammalian mitochondrial integrity in a context-dependent fashion, and this has profound implications for our molecular understanding of vertebrate mitophagy.**

## INTRODUCTION

Recently, research has elaborated the mechanisms by which two enzymes, PTEN-induced kinase 1 (PINK1) and Parkin, mediate a mitochondrial quality control pathway, which leads to removal of damaged mitochondria by selective autophagy (mitophagy; McWilliams and Muqit, 2017). Mitochondrial damage activates the mitochondrial-associated kinase PINK1, which in turn phosphorylates both ubiquitin and the cytosolic E3-ubiquitin ligase Parkin at their respective Ser65 residues. This initiates a feedforward amplification cascade of mitochondrial ubiquitylation that drives clearance of the damaged organelle via mitophagy (Ordureau et al., 2014). As mutations in human PINK1 (*PARK6*) and Parkin (*PARK2*) are causative for early-onset Parkinson's disease (PD), aberrant mitophagy has emerged as an attractive hypothesis to explain the pathophysiology of this neurodegenerative movement disorder (McWilliams and Muqit, 2017; Whitworth and Pallanck, 2017). Although PINK1 is regarded as a master regulator of mitophagy, little is known about its exact contribution to mammalian mitophagy *in vivo*. This paucity of data is largely due to the difficulty of studying both endogenous PINK1 signaling and mitophagy in mammalian tissues. Furthermore, the majority of studies on PINK1-dependent mitophagy have been conducted using overexpressed proteins and mitochondrial uncoupling agents that artificially induce depolarization in cultured cells, creating a conspicuous asymmetry between observations of PINK1-mediated mitochondrial clearance *in vitro* and its relevance to the regulation of mitophagy *in vivo*. We recently demonstrated the striking nature of basal mitophagy *in vivo* using the *mito*-QC mouse model, a fluorescence-based reporter, which enables the subcellular visualization of mitochondrial architecture and mitophagy in tissues (McWilliams and Ganley, 2016; McWilliams et al., 2016). To interrogate the function of PINK1 in regulating basal mitophagy *in vivo*, we characterized *Pink1* wild-type (WT) and knockout (KO) mice expressing the *mito*-QC transgene, focusing on tissues of high metabolic dependence and cell types of clinical importance to PD. We report basal mitophagy in subsets of highly metabolic cells *in vivo*, including dopaminergic (DA) neurons, retinal photoreceptor neurons, and exocrine pancreatic acinar cells. Furthermore, across a range of quantitative parameters, we demonstrate that basal mitophagy *in vivo* occurs independently of PINK1 in a variety of tissues. Our findings are further substantiated by the biochemical detection and validation of endogenous PINK1 within tissues. These findings broaden our understanding of mitochondrial homeostasis *in vivo,* suggesting undiscovered regulators and mechanisms.

## RESULTS

### *mito*-QC Reports Endogenous PINK1-Dependent and Independent Stress-Evoked Mitophagy

We previously validated *mito*-QC as a *bona-fide* endpoint mitophagy reporter in cultured cells and tissues (Allen et al., 2013; McWilliams et al., 2016). To assess the contribution of mammalian PINK1 to basal mitophagy *in vivo*, we crossed *Pink1* KO and *mito*-QC reporter mice to obtain *Pink1* WT


This is an open access article under the CC BY license (http://creativecommons.org/licenses/by/4.0/).

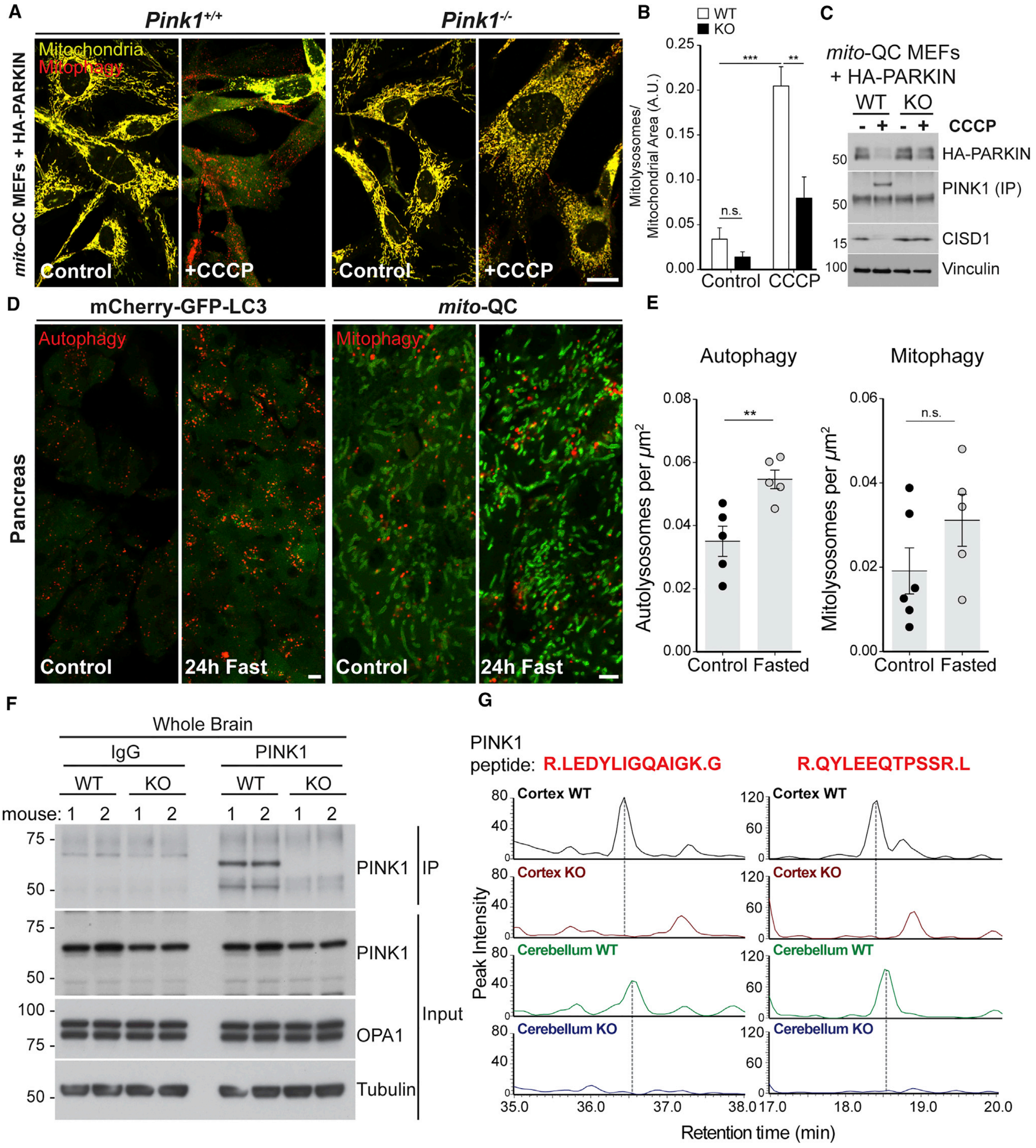


**Figure 1. Detection of PINK1-Dependent Mitophagy *In Vitro* and Detection of Endogenous PINK1 Protein at Basal Levels *In Vivo***

(A) Primary MEF cultures established from *Pink1*$^{+/+}$ *mito*-QC$^{+/+}$ and *Pink1*$^{-/-}$ *mito*-QC$^{+/+}$ littermate embryos were transduced with retroviral HA-Parkin, and stimulated with DMSO (Control), CCCP, or DFP for 24 hr. Mitophagy (mCherry-only mitolysosomes) was visualized by confocal microscopy.

(B) Mitophagy was readily observed in CCCP-treated WT and not in KO MEFs (two-way ANOVA, Bonferroni correction: ***$p < 0.001$, **$p < 0.01$; n.s., $p > 0.05$).

(C) Immunoblot showing levels of exogenous HA-Parkin and endogenous PINK1 protein following CCCP treatment in *Pink1* WT and KO *mito*-QC MEFs. Diminished levels of the Parkin substrate CISD1 and equal loading using vinculin are shown.

(D) Pancreas sections from mCherry-GFP-LC3 mice and *mito*-QC animals from fasting experiments.

(E) Elevated levels of general autophagy are observed following a 24-hr fasting period, compared with animals fed *ad libitum* (Student's t test; **$p < 0.01$). A corresponding induction of mitophagy is not observed upon starvation (n.s., $p > 0.05$).

*(legend continued on next page)*

(*Pink1*$^{+/+}$) and KO (*Pink1*$^{-/-}$) mice on a homozygous *mito*-QC background. Resultant offspring were born at normal Mendelian frequencies and exhibited no obvious physical or anatomical abnormalities. To verify that our system faithfully detects PINK1-dependent mitophagy, we established primary mouse embryonic fibroblast (MEF) cultures from heterozygote matings of *Pink1*$^{+/-}$ *mito*-QC$^{+/+}$ animals. We transduced the resulting *Pink1* WT and KO littermate cultures with retroviral HA-Parkin, and treated MEFs with the mitochondrial uncoupler carbonyl cyanide 3-chlorophenylhydrazone (CCCP). Consistent with published observations (Narendra et al., 2008), *mito*-QC revealed the characteristic elimination of depolarized mitochondria in *Pink1* WT, but not *Pink1* KO MEF cultures (Figures 1A and 1B). This was associated with clear stabilization of PINK1 in CCCP-treated WT cells only, as determined by immunoprecipitation (IP) and immunoblot analysis of endogenous PINK1 (Figure 1C). In parallel, we observed loss of Parkin expression (due to autoubiquitylation upon activation) in WT, but not in KO *mito*-QC MEFs treated with CCCP (Figure 1C). We previously demonstrated that iron-chelation-induced mitophagy occurs independently of PINK1/Parkin (Allen et al., 2013). Consistent with this, *mito*-QC demonstrated deferiprone-induced mitophagy in both PINK1 WT and KO MEF cultures (Figures S1A and S1B). Our experiments further confirmed that endogenous PINK1 activates HA-Parkin E3 activity, as demonstrated by diminished levels of its OMM substrate CISD1 in *Pink1* WT, but not KO *mito*-QC MEFs (Figure 1C). In cultures of primary mouse adult fibroblasts (non-*mito*-QC) treated with CCCP, we also observed ubiquitylation of CISD1 by endogenous Parkin and generation of phospho-ubiquitin in *Pink1* WT, but not KO samples (Figure S1C). Taken together, our data demonstrate that *mito*-QC reliably reports both PINK1-dependent and independent forms of stress-evoked mitophagy.

To provide additional verification that *mito*-QC monitors the selective autophagy of mitochondria *in vivo*, we generated another fluorescence-based reporter mouse to visualize general macroautophagy based on the same principle. This model was generated by knock in of mCherry-GFP-LC3 (*mCherry-GFP-Map1lc3b*) at the *Rosa26* locus, as for *mito*-QC (Figure S1D). Under normal conditions, the reporter displays a diffuse cytosolic staining pattern, but is recruited to autophagosomes during autophagy, which appear both green and red. Upon autophagosome-lysosome fusion, GFP fluorescence is quenched and mCherry-only puncta serve as markers of resultant autolysosomes. Using both mitophagy and autophagy reporter mouse models, we performed the classical starvation experiment previously described by the Mizushima laboratory (Mizushima et al., 2004). After a 24-hr fasting period, we observed elevated macroautophagy in the pancreas as described previously (Figures 1D and 1E). Notably, we did not detect differences in mitophagy between fasted and non-fasted animals (pancreatic mitophagy is detailed in Figure 4 and discussed thereafter). These data show that under identical starvation conditions, *mito*-QC selectively reports mitophagy, demonstrating the specificity of the reporter as well as highlighting the differing requirements that regulate mitophagy versus starvation-induced autophagy.

In cell culture, under normal conditions, PINK1 is undetectable due to rapid turnover via constitutive N-end rule degradation (Yamano and Youle, 2013). As PINK1 only becomes stabilized and activated upon mitochondrial depolarization, it has been widely assumed that PINK1 would not be detectable at basal levels in normal, healthy tissue. This view is further entrenched by a lack of tools that facilitate the robust detection of mouse PINK1 at basal levels *in vivo*, which can also be verified by essential KO controls. Our biochemical MEF data demonstrate that we can detect endogenous PINK1 using two in-house generated antibodies (Figure 1C). Thus, we investigated if PINK1 is detectable at basal levels within tissues, notably in the CNS. We performed IPs from total brain extracts from *Pink1* WT and KO mice. We were able to clearly detect a 60-kDa band corresponding to mouse PINK1 in WT brain lysates, which was absent in the KO samples (Figure 1F). We employed mass spectrometry (MS) to obtain gold-standard analytical verification of our biochemical results (Figures 1G and S1E). Satisfyingly, MS analysis revealed peptides corresponding to PINK1 in samples from WT but not KO mice (Figures 1G and S1E). Our rigorous approach provides conclusive evidence that PINK1 protein is present in the mouse brain under normal physiological conditions.

## *Pink1* Is Dispensable for Basal Neural Mitophagy *In Vivo*

We next sought to obtain a spatial snapshot of PINK1 expression in the mammalian nervous system. To do this, we microdissected neural substructures from *Pink1* WT and KO adult mouse brains, obtained protein lysates, and used our validated biochemical IP approach to detect PINK1. We observed PINK1 expression across all CNS regions, with particular enrichment in the striatum, neocortex, cerebellum, and spinal cord. Interestingly, we detected lower levels of PINK1 in the olfactory bulbs (Figure 2D). This suggests that PINK1 may undergo differential processing at different rates within distinct regions of the adult brain.

Neural tissue has a very high energy demand, and maintenance of mitochondrial homeostasis is essential to sustain this. Aberrant mitochondrial quality control is predicted to underlie several neurodegenerative disorders, including PD, which is characterized by the progressive loss of A9 DA nigrostriatal projection fibers (Grealish et al., 2010; Poewe et al., 2017). Although impaired mitophagy has been proposed as a main contributor to DA neurodegeneration, evidence as to whether DA neurons undergo basal mitophagy *in vivo*, and to what degree, has remained elusive. We thus performed comparative analyses of mitophagy in brain sections from *Pink1* WT and KO *mito*-QC mice, where total brain extracts showed that the *mito*-QC reporter was similarly expressed in both *Pink1* WT and KO animals, and that mitochondrial protein expression was comparable (Figure S2A).

(F) IP-immunoblot showing biochemical detection of endogenous PINK1 protein in mouse brain homogenates, n = 2 animals per genotype. PINK1 protein is detectable at basal levels using enrichment by IP, but not in whole-cell lysates (inputs). Note the absence of the PINK1 band in the immunoglobulin G (IgG) control and KO samples.

(G) Analytical validation of PINK1 biochemical experiments by mass spectrometry. Example of extracted-ion chromatograms detailing two unique PINK1 peptides present in IP's from WT, but not PINK1 KO cortex and cerebellum. Data are represented as mean ± SEM.

Scale bars, 5 μm. See also Figure S1.

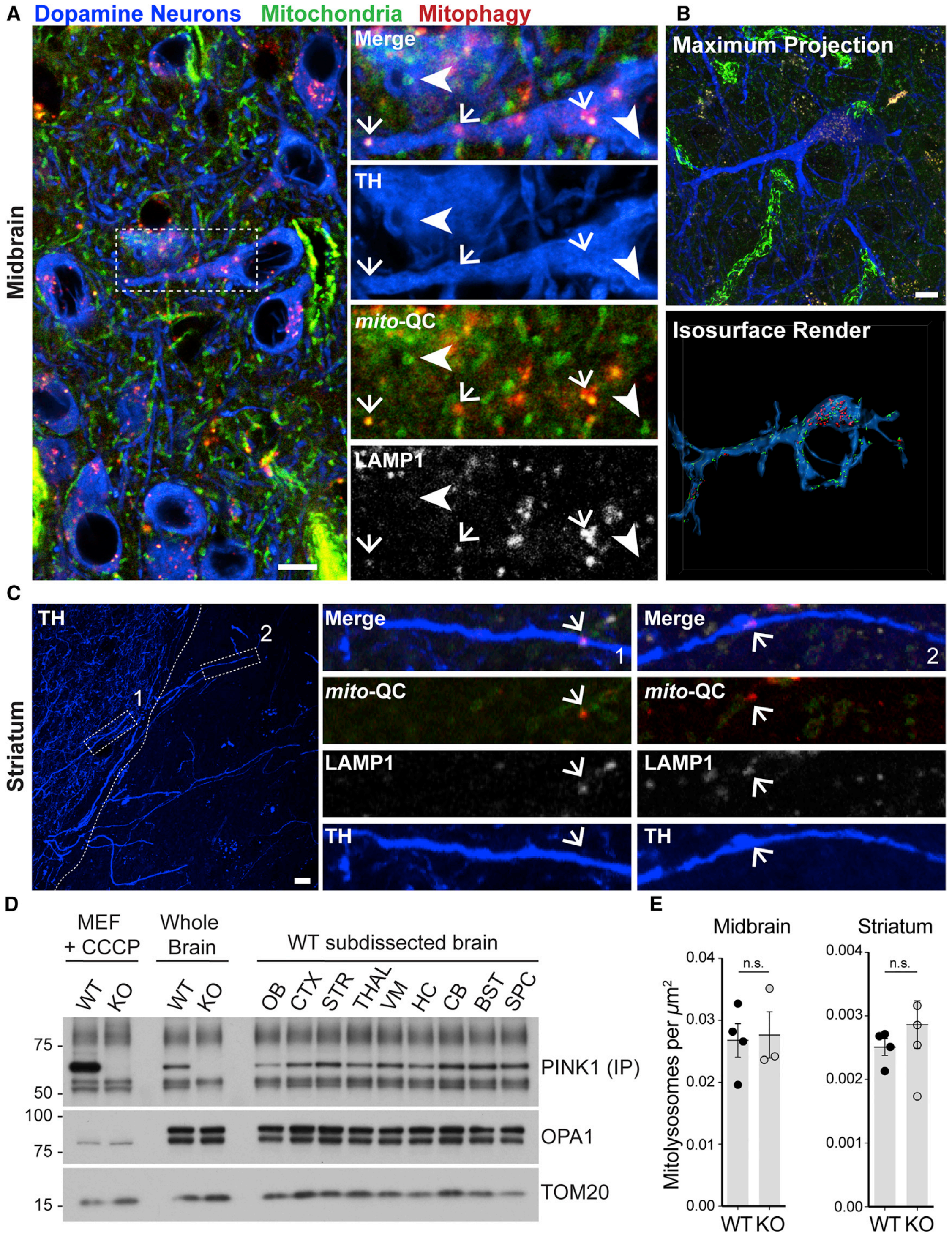


**Figure 2. Basal Mitophagy Proceeds in the Mammalian Dopaminergic System *In Vivo* in the Absence of *Pink1***

(A) Representative micrograph showing immunolabeled midbrain dopaminergic neurons undergoing basal mitophagy *in vivo*. Sections of *mito*-QC mouse ventral midbrain were labeled with antibodies to the DA marker tyrosine hydroxylase (TH) and LAMP1 (inset: arrows indicate mitolysosomes and arrowheads show mitochondria).

(B) Collapsed *Z* projection of a midbrain dopaminergic neuron with corresponding isosurface render (below), detailing 3D localization of mitolysosomes.

(C) Instances of axonal mitophagy in nigrostriatal DA projections are less frequent compared with DA somata. Low-power micrograph showing region of dorsolateral striatum revealed by TH. Panels show instances of axonal mitophagy (arrows, 1 and 2), where LAMP1-positive mitolysosomes are detected within terminal DA axons *in vivo*.

*(legend continued on next page)*

Midbrain DA (mesDA) neurons were distinguished using the catecholaminergic marker, tyrosine hydroxylase (TH), to identify cell-specific mitophagy. Given the ontogeny of nigrostriatal projections, transverse brain sections were employed to investigate mitophagy in somata, axonal projections, and terminal arbors of the entire pathway. This orientation also enabled a valuable comparative assessment of mitophagy in other DA populations, such as the adjacent A10 DA neurons of the ventral tegmental area, and A11 DA neurons of the periventricular hypothalamus. Intriguingly, we observed high levels of mitophagy in the somata of TH-positive nigral DA neurons (Figures 2A, 2B, and S2B). LAMP1-immunostaining confirmed the lysosomal origin of morphologically distinct mitolysosomes within DA neurons (Figures 2A and 2C). The reporter signal also revealed the somata of TH-positive DA neurons to have a low mitochondrial mass, consistent with previously published electron microscopic studies (Liang et al., 2007). Maximum intensity projections combined with volume surface rendering of immunostained structures enabled us to visualize the spatial nature of nigrostriatal mitophagy in 3D (Figures 2B and S2B). Although we observed minimal turnover in DA processes, we detected several instances of mitophagy enriched in, or proximal to, the axon initial segments/hillocks of DA neurons. These findings suggest that mitophagy within DA neurons is likely to be a dynamic process *in vivo*. Although we found limited numbers of mitolysosomes within DA terminal arbors (Figure 2C), we cannot preclude a model of retrograde transport where mitophagosomes formed in distal DA compartments, may fuse with lysosomes at a region proximal to or within the somata. Alternatively, it is also plausible that the enrichment of mitolysosomes in DA somata may arise from the destruction of local mitochondria. This could represent a distinct mitochondrial QC checkpoint in neurons, whereby constitutive mitochondrial-damage surveillance in the cell body prevents defective mitochondria from entering axons. Further work will be needed to clarify this. Our data demonstrate a close correlation between the high levels of DA mitophagy and the previously reported high metabolic activity of these cell types (Pacelli et al., 2015). In all instances, basal mitophagy was indistinguishable between *Pink1* WT and KO animals (Figures 2E and S2C).

We extended our analysis to another PD-related DA population of clinical interest, the rostral A16 periglomerular DA neurons (PGNs) of the olfactory bulb. Olfactory dysfunction, in particular hyposmia, affects 90% of all PD patients and is a well-established pre-motor symptom, although the mechanisms are not fully understood (Doty, 2012b; Markopoulou et al., 1997; Ponsen et al., 2004). PGNs mediate lateral inhibition of mitral neurons within the olfactory bulb (Martinez and Freeman, 1984) and in contrast to the selective degeneration of A9 neurons, PGNs are known to double in number in human PD patients and in pre-clinical neurotoxin models of PD (Huisman et al., 2004). As with the A9 and A10 population of DA neurons, we observed robust levels of mitophagy in PGNs, with most turnover confined to somata (Figure S2D). Morphologically, PGNs are described as "axonless" ultrashort projection neurons (Doty, 2012a). Similar to the other DA populations we assessed, both *Pink1* WT and KO PGNs exhibited comparable levels of mitophagy (Figure S2E). 3D isosurface rendering revealed the complex associations of olfactory DA neurons and mitochondrial-rich glomeruli (Figures S2D–S2F), where olfactory receptor neurons synapse with PGNs. Surprisingly, little mitophagy is observed in this area, consistent with our observations that most DA neuronal mitophagy occurs within somata. We also explored mitophagy in diencephalic DA neurons. These DA neurons undergo a lower degree of mitophagy than other subtypes we assessed, yet no differences were observed between *Pink1* WT and KO neurons (data not shown). As we can detect PINK1 protein in the nervous system and basal mitophagy in the DA neurons, our results demonstrate that loss of PINK1 does not perturb basal neuronal mitophagy *in vivo*.

## Basal Mitophagy in Non-neuronal Cells Occurs Independently of *Pink1*

Microglia have emerged as important mediators of development and neuroinflammation in the vertebrate nervous system. Furthermore, PINK1 has been reported to influence the function of these non-neuronal cells (Flinn et al., 2013; Kim et al., 2013; Mondelli et al., 2017; Perry and Holmes, 2014). We labeled sections with anti-Iba1 to assess mitophagy within microglia *in vivo*. We detected basal mitophagy within cortical and hippocampal-resident Iba1-positive microglia (Figure 3A), visualization of which was facilitated by 3D rendering (Figure 3B). Comparative analysis revealed no pronounced differences in microglial mitophagy between *Pink1* WT and KO mice (Figures 3C, 3D, and S3C).

A consistent observation during our studies has been the intimate association of cerebral vasculature with neurons and neuroglia, which was highlighted by the prominent expression of the *mito*-QC reporter within the vascular network. The relationship between cerebral vasculature, neural activity, and metabolism are well documented, and the study of cerebrovascular mitochondria has major translational implications for several neurological disorders (Kluge et al., 2013; Zlokovic, 2011). Although the neurogliovascular complex is a fundamental unit of the nervous system, it is very difficult to study the integrative mitochondrial biology of such complex associations *in vitro*. In *mito*-QC brain sections, we easily resolved individual mitochondria in blood vessels, and consistently observed robust levels of vascular mitophagy (Figures 3E and 3F). PINK1 has also been reported to function in vasculature, yet neurovascular mitophagy was comparable between *Pink1* WT and KO *mito*-QC animals (Figures 3G and S3C). We also observed evidence for mitophagy

(D) IP-immunoblot showing rostro-caudal expression pattern of PINK1 protein in the adult mouse nervous system under basal conditions. PINK1 is differentially expressed in lysates of sub-dissected adult mouse brain. OB, olfactory bulb; CTX, neocortex; STR, striatum; Thal, thalamus; VM, ventral midbrain; HC, hippocampus; CB, cerebellum; BST, brainstem; SPC, spinal cord. CCCP-treated MEFs and total brain homogenates from *Pink1* WT and KO demonstrate the specificity of the endogenous PINK1 band.

(E) Analyses of mitophagy in the nigrostriatal system of *Pink1* WT and KO animals reveal no differences between genotypes (Student's t test; n.s., $p > 0.05$). Data are represented as mean ± SEM.

Scale bars, 5 μm. See also Figure S2.

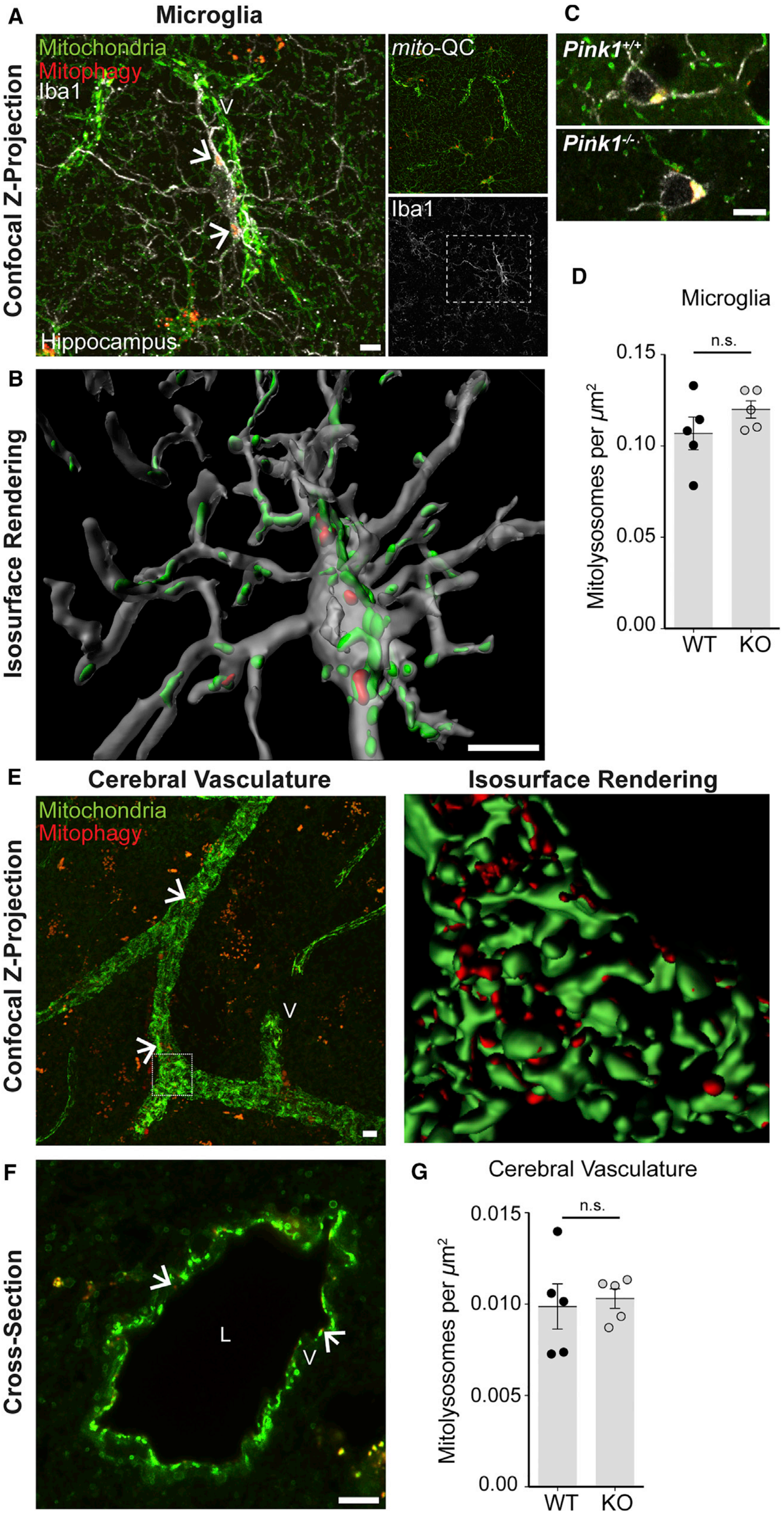


**Figure 3. Microglia and Cerebrovascular Mitophagy Occurs Independently of *Pink1 In Vivo***

(A) Iba1-positive microglia exhibit robust mitochondrial clearance *in vivo*. Confocal photomicrograph shows microglial cell undergoing mitophagy intimately associated with vasculature (v). Arrows indicate mitolysosomes within microglia.

(B) Isosurface rendering highlights the 3D spatial arrangement of microglial mitochondria and mitolysosomes *in vivo*.

(C) Representative images of microglial mitophagy in *Pink1* WT and KO mice.

(D) Quantitation of microglial mitophagy.

(E) Representative image showing basal mitophagy in adult striatal vasculature *in vivo* with associated rendered 3D projection detailing mitochondrial architecture. Arrows indicate examples of mitolysosomes.

(F) Airyscan cross-section of a striatal blood vessel details arrangement of vascular mitochondria and mitolysosomes *in vivo*. L indicates lumen and V denotes vessel. Arrows indicate examples of mitolysosomes.

(G) Quantitation of cerebrovascular mitophagy in *Pink1* WT and KO mice (Student's t test; n.s., $p > 0.05$). Data are represented as mean ± SEM.

Scale bars, 5 μm. See also Figure S2.

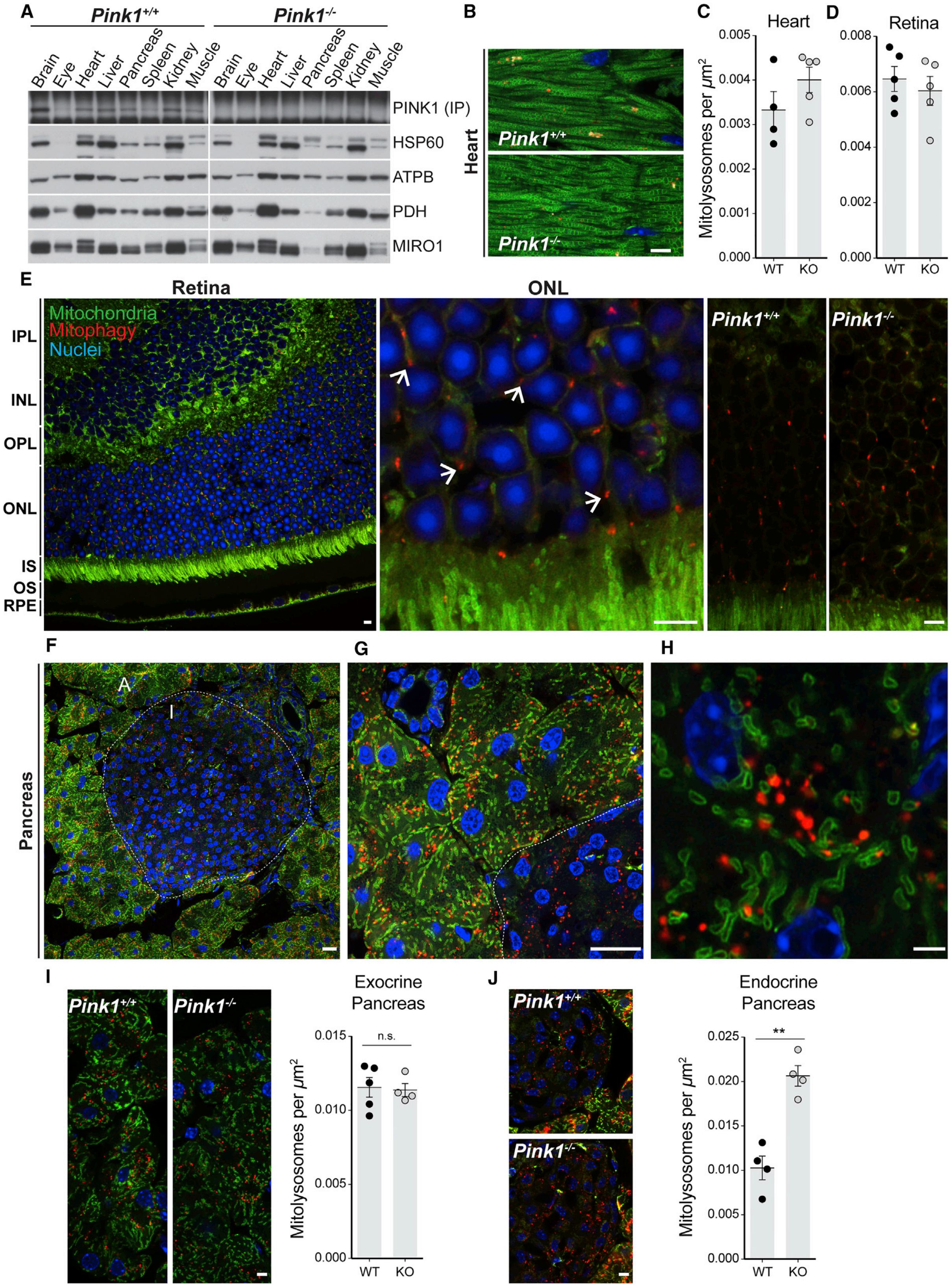


(legend on next page)

in GFAP-positive astrocytes *in vivo* (Figure S2G). Our data unearth the selective degradation of mitochondria in mammalian microglia and vasculature *in vivo*, and show that basal mitophagy proceeds in the absence of *Pink1* in non-neuronal cells. These data will be important to better understand mitochondrial QC in neuroinflammatory and cerebrovascular diseases.

## PINK1-Independent Basal Mitophagy in Tissues of High Metabolic Demand

As we could detect PINK1 protein at basal levels in the nervous system, we next assayed its expression across a range of metabolically active tissues (Figure 4A). PINK1 was expressed in the brain as well as in the heart, liver, kidney, and pancreas (Figure 4A). The metabolic demands of the heart are well described, and *Pink1* has been proposed to play key functions in cardiac mitochondrial homeostasis (Billia et al., 2011). However, a comparative assessment of mitophagy in *Pink1* WT and KO adult heart sections revealed no differences in the number of cardiac mitolysosomes (Figures 4B and 4C). Furthermore, assessment of mitochondrial morphology obtained from the GFP channel, revealed no differences in the animals we assessed (Figure S3C). We next extended our analysis of mitophagy to other tissues of high metabolic dependence. We and others have previously reported high levels of hepatic mitophagy (McWilliams et al., 2016; Menzies and Gold, 1971). Upon investigation of liver sections from *Pink1* WT and KO mice we found no overt differences in levels of mitophagy between genotypes (data not shown).

A constituent tissue of the CNS, the retina has been suggested to be one of the most metabolically active tissue in mammals (Ames et al., 1992; Wong-Riley, 2010). The eye provides a contrast, as PINK1 protein expression was noticeably lower than in other tissues (Figure 4A). We observed a striking tissue-specific restriction of ocular mitophagy to the retinal outer nuclear layer (ONL), which contains the cell bodies of the rod and cone photoreceptor cells (Figure 4E). Airyscan microscopy revealed an elaborate mitochondrial network in the retina, with the majority of mitolysosomes confined to the ONL (Figure 4E). Associated striated rectus muscle on tissue sections exhibited comparable levels of mitophagy (Figure S3A). As with other tissues assessed, loss of *Pink1* did not alter basal retinal mitophagy (Figures 4D and 4E).

During the course of our investigations we observed pronounced mitophagy in pancreatic acinar cells, which are responsible for enzymatic and electrolyte release into the gastrointestinal tract. The pancreas is an organ of major clinical importance due to its dysfunction in inflammatory, neoplastic, and endocrine disease (Gerasimenko and Gerasimenko, 2012). The OMM-localization of *mito*-QC facilitated the visualization of this unique mitochondrial network with ease (Figures 4F–4H). Acinar cells contained elongated and highly tubular stereotypically shaped mitochondria (Figures 4G and 4H). Three distinct mitochondrial subgroups have been suggested to modulate pancreatic acinar cell function (Johnson et al., 2003; Park et al., 2001; Petersen, 2012). These classical discoveries were made on isolated primary acinar cells and electron microscopy studies of pancreatic acinar cells *in vivo*. Using *mito*-QC reporter mice, we could resolve sub-plasmalemmal, perinuclear, and perigranular mitochondria in sections of intact pancreas (Figure S3D). These distinct mitochondrial subpopulations are known to buffer intracellular calcium signaling, as well as synchronizing ATP supply to metabolic demand (Gerasimenko and Gerasimenko, 2012; Johnson et al., 2003; Petersen, 2012). Interestingly, a recent publication describes the importance of macroautophagy to pancreatic acinar homeostasis (Antonucci et al., 2015). Although PINK1 is expressed in human exocrine pancreas (PARK6; Human Protein Atlas), and loss of Parkin has been linked to pancreatic tumorigenesis (Sun et al., 2013; Uhlen et al., 2015), we found no differences between WT and KO *Pink1 mito*-QC animals (Figure 4I). In contrast to acinar cells, less mitophagy was observed in islets (Figures 4F and 4G). However, analysis of endocrine islet cells showed an unexpected elevation of mitophagy in *Pink1* KO animals compared with WT (Figure 4J). It has previously been shown that *Pink1* deficiency in pancreatic β-cells results in impaired metabolism due to mitochondrial dysfunction (Deas et al., 2014). Our discovery of elevated mitophagy in *Pink1* KO islets are consistent with mitochondrial impairment and suggest that, in this scenario, PINK1-independent mitophagy can be upregulated. This hints that in addition to mitophagy being decreased during certain disease states (Sun et al., 2015), elevated mitophagy may also contribute to metabolic pathophysiology. Together, our data demonstrate that PINK1 is dispensable for basal mitophagy in a variety of tissues, and the degree of mitophagy depends on metabolic context. Future work will be essential to determine the molecular basis of PINK1-independent mitophagy in the pancreas, and its clinical significance in PD patients.

**Figure 4. Profiling PINK1 Expression and Basal Mitophagy in Tissues of High Metabolic Demand Using *Pink1* WT and KO Mice**

(A) Comparative analysis of PINK1 expression *in vivo*. PINK1 is differentially expressed in a range of metabolically active tissues.

(B and C) Normal cardiomitophagy in *Pink1* WT and KO animals *in vivo*.

(D) Quantitation of retinal mitophagy between genotypes. Student's t test; n.s., $p > 0.05$.

(E) Mammalian retinal mitophagy *in vivo*. Photomicrograph of adult retina in a section of adult eye from the *mito*-QC mouse. Individual layers are demarcated by counterstained nuclei (Hoescht) and *mito*-QC expression. IPL, inner pexiform layer; INL, inner nuclear layer; OPL, outer plexiform layer; ONL, outer nuclear layer; IS, inner segment; OS, outer segment; RPE, retinal pigmented epithelium. Shown is an Airyscan image detailing mitophagy in a region of the ONL adjacent to the IS and representative images demonstrating that loss of *Pink1* does not alter levels of basal mitophagy in the retinal ONL. Arrows indicate examples of mitolysosomes.

(F) Mitophagy and mitochondrial networks of pancreatic acinar cells "A" and islets "I" *in vivo*.

(G) Airyscan microscopy details pancreatic mitophagy within exocrine acinar cells and an adjacent islet.

(H) High-resolution Airyscan image showing individual mitochondria, mitolysosomes, and nuclei of pancreatic acinar cells *in vivo*.

(I) Representative confocal micrographs showing pancreatic mitophagy in *Pink1* WT and KO *mito*-QC animals. Quantitation of exocrine pancreatic mitophagy between genotypes (Student's t test; n.s., $p > 0.05$).

(J) Representative confocal micrographs of islet mitophagy in *Pink1* WT and KO *mito*-QC animals. Quantitation of endocrine mitophagy between genotypes. Data are represented as mean ± SEM. $^{**}p < 0.01$.

Scale bars, 5 μm. See also Figures S3 and S4.

Although *mito*-QC can report stimulus-induced mitophagy *in vitro*, we wanted to determine if the same was true *in vivo*. Mitophagy has been typically studied in the context of *in vitro* mitotoxicity, yet its role as a neuroprotective pathway remains unclear. Thus, we assessed if mitophagy is induced under conditions of chronic mitochondrial neurotoxicity *in vivo* using the well-studied brain-penetrant mitotoxin, 3-nitroproprionic acid (3-NPA), which irreversibly inhibits complex II of the electron transport chain (Beal et al., 1993; Brouillet et al., 2005). Intraperitoneal administration of 3-NPA selectively induces striatal lesions *in vivo*, although its variability in terms of resistance and lesion heterogeneity is well documented. Lesion studies using 3-NPA have also been shown to damage cerebral vasculature and mitochondrial-rich peripheral organs. Using a 7-day systemic administration of 3-NPA in *Pink1* WT and KO *mito*-QC animals (n = 6 WT and n = 9 KO animals per 3-NPA group), we observed a small number of heterogeneous and variable intrastriatal lesions with elevated mitophagy; however, overall levels of striatal mitophagy were unchanged between groups (Figure S4A). 3-NPA also induces damage to vascular endothelial cells and increases blood-brain barrier extravasation in the striatum (Duran-Vilaregut et al., 2009; Nishino et al., 1995), and the present study describes robust levels of mitophagy in cerebrovasculature. We did not find any effect of 3-NPA that could influence mitophagy in *Pink1* WT and KO striatal vasculature (Figure S4A). To determine if 3-NPA could in fact activate the canonical PINK1-Parkin pathway, we analyzed endogenous signaling in primary MEFs. PINK1 protein was stabilized in WT MEFs treated with CCCP, but not 3-NPA (Figure S4B). This is consistent with previous findings in HEK293 cells (Kondapalli et al., 2012). 3-NPA is also known to induce peripheral pathology in mitochondrial-rich organs such as the heart, yet cardiac mitophagy was comparable between 3-NPA-treated WT and KO animals (Figure S4A). Assessment of skeletal muscle provided a unique opportunity to interrogate *Pink1* function in mitophagy during different metabolic contexts, as the soleus muscle is highly oxidative, whereas the gastrocnemius is progressively more glycolytic as distance increases from the soleus. We observed no differences in the number of mitolysosomes between *Pink1* WT and KO animals at basal levels or in those treated with 3-NPA (data not shown). Interestingly, we observed a trend towards elevated mitochondrial content in 3-NPA-treated *Pink1* KO animals, particularly in the more oxidative muscle, signifying an adaptive response to stress (Figures S4C and S4D). Although the total mitochondrial signal increased, the corresponding degree of mitophagy did not. Hence, when we analyzed mitophagy as a function of increased mitochondrial content, we observed a small, yet consistent, reduction in mitophagy in oxidative and glycolytic muscle regions in *Pink1* KO animals treated with 3-NPA (Figure S4E). Our findings indicate that, in the context of *in vivo* mitophagy, the function of PINK1 is cell-specific and highly dependent upon metabolic context.

## DISCUSSION

Our work reveals the *in vivo* prevalence of mitophagy in a variety of organ systems and cell types of high metabolic demand. We sought to address a longstanding question of major importance in this field, by assessing the contribution of PINK1 to the regulation of basal mitophagy *in vivo*. We were surprised to find that *Pink1* KO mice exhibited indistinguishable basal mitophagy compared with WT animals. Apart from a modest, yet consistent, context-dependent alteration in mitophagy levels in endocrine pancreas and skeletal muscle, our multi-parametric analyses did not reveal any overt differences in the number, shape, size of mitolysosomes in tissues, or in mitochondrial morphology across the vast majority of tissues assessed. Our demonstration of endogenous PINK1 protein expression, at basal levels within a range of major organ systems, strengthens our observations in *mito*-QC animals. Furthermore, our approach provides a long-awaited and rigorous framework for the biochemical detection of mouse PINK1 protein *in vivo*. Coupled with the recent discovery of the PINK1 X-ray crystal structure, our methods will be important for assessing PINK1 activation using small-molecule compounds *in vivo* (Kumar et al., 2017). Overall, we do not conclude that PINK1 is dispensable for all forms of mitophagy *in vivo*. Rather, we assert that distinct basal and stress-evoked pathways exist to co-ordinate mitochondrial clearance in a context-dependent fashion. Our data suggest that the precise role of PINK1 *in vivo* remains to be defined.

Since the landmark discovery of Parkin-dependent mitochondrial clearance (Narendra et al., 2008), the PINK1-Parkin pathway has quite rightly dominated the field of mitophagy (McWilliams and Muqit, 2017; Pickrell and Youle, 2015). The clinical significance of this pathway is irrefutable, yet our data show that, *in vivo*, it is not the major pathway that regulates basal mitophagy. Although our data derives from a mouse model, it is reasonable to assume an analogous scenario in humans. PD patients survive in the absence of a functional PINK1-dependent mitophagy pathway, and primary locomotor symptoms do not usually manifest until the second and third decades of life. We speculate that a complete blockade in mitophagy would confer a more severe phenotype.

We offer two potential explanations that may account for our observations of PINK1-independent basal mitophagy *in vivo*. (1) Mammalian cells harbor multiple mitophagy pathways that can be triggered in response to diverse stimuli under certain conditions. Therefore it is conceivable that the activation of PINK1-dependent mitophagy is highly context-dependent in mammals, and operates in response to a distinct type of stress. Thus, overt differences may only become apparent between *Pink1* WT and KO mice under extreme conditions of chronic mitotoxicity or aging. Previously, it has been shown that loss of endogenous *Parkin* triggers age-dependent DA neurodegeneration under conditions of chronic mitotoxicity in mice (Pickrell et al., 2015). However, in this study, DA neurodegeneration resulting from loss of endogenous *Parkin* in the *Polg*$^{D257A}$ background was not ascribed to dysregulated mitophagy, due to the already complex pathological background of *mutator* mice (Trifunovic et al., 2004). (2) We studied animals generated by traditional transgenesis, i.e., germline ablation of the *Pink1* gene. Thus, developmental or epistatic compensation could account for the largely normal or unperturbed mitochondrial homeostasis observed in *Pink1* KO *mito*-QC animals. This was elegantly demonstrated for *Parkin* in studies from the Dorn and Chan laboratories (Gong et al., 2015; Rojansky et al., 2016). In the Dorn study, conditional deletion of *Parkin* during perinatal myocardial development caused the onset of severe cardiomyopathy (Zhang

et al., 2008). In the Chan study, MUL1 activity was demonstrated to operate in a parallel pathway to Parkin, potentially explaining why *Parkin* KO mice do not recapitulate overt neurodegenerative phenotypes (Rojansky et al., 2016). Accordingly, more sophisticated mouse genetics may be required to observe any dramatic effect of PINK1 on basal mitophagy *in vivo*. Naturally, our results describing PINK1-independent mitophagy should provoke a reassessment of the precise function of PINK1 *in vivo*. However, such an investigation extends beyond the scope of this current report.

That mitophagy proceeds in the absence of PINK1 demonstrates that very little is known about the molecular mechanisms of basal mitophagy *in vivo*, and that these data should stimulate much needed work in this area. Our data should also provoke debate on multiple aspects of PD pathophysiology with respect to mitophagy. Key questions still remain as to when and where stimulus-evoked PINK1-dependent mitophagy is required *in vivo*, as this may help determine much needed therapeutic strategies for PD. Ultimately, it will be vital to identify key regulators of cell and tissue-specific basal mitophagy *in vivo*.

## STAR★METHODS

Detailed methods are provided in the online version of this paper and include the following:

- KEY RESOURCES TABLE
- CONTACT FOR REAGENT AND RESOURCE SHARING
- EXPERIMENTAL MODEL AND SUBJECT DETAILS
  - Animals
  - *In Vitro* Models
- METHOD DETAILS
  - Stimulation of Mitophagy
  - Histology, Immunochemistry & Confocal Microscopy
  - Tissue Harvesting and Preparation of Protein Lysates
  - Immunoblotting
  - Detection of Endogenous PINK1 Protein in Mouse Tissues
  - Detection of Endogenous Mouse PINK1 Protein in Brain Regions by Mass Spectrometry
- QUANTIFICATION AND STATISTICAL ANALYSIS
  - Semi-Automated Quantitation
  - Statistical Analysis

## SUPPLEMENTAL INFORMATION

Supplemental Information includes four figures and can be found with this article online at https://doi.org/10.1016/j.cmet.2017.12.008.

## ACKNOWLEDGMENTS

We thank Rachel Hills, David Campbell, Fiona Brown, and Axel Knebel for technical assistance. We thank Miguel Martins (Leicester) for cryopreserved *Pink1* KO embryos. We thank support teams and colleagues in Biological Services (Dundee and Cardiff) for genotyping and animal husbandry. This work was funded by grants from The Michael J. Fox Foundation for Parkinson's disease research (11470 to I.G.G. and M.M.K.M.), the Medical Research Council, UK (MC_UU_12016/4 to I.G.G.), and Wellcome (101022/Z/13/Z to T.G.M., E.B., and M.M.K.M.). G.B. is supported by an MRC Next Generation Optical Microscopy award (MR/K015869/1), and Dundee Imaging Facility is supported by the "Wellcome Trust Technology Platform" award (097945/B/11/Z).

## AUTHOR CONTRIBUTIONS

Conceptualization, I.G.G. and T.G.M.; Methodology, I.G.G., T.G.M., and S.P.B.; Investigation, T.G.M., A.R.P., L.M.-G., F.S., E.B., and S.P.B.; Formal Analysis, G.B. and F.S.; Writing – Original Draft, T.G.M. and I.G.G.; Writing – Reviewing & Editing, T.G.M., I.G.G., A.R.P., L.M.-G., and F.S.; Funding Acquisition, I.G.G. and M.M.K.M.; Supervision, I.G.G. and M.M.K.M.

Received: May 21, 2017
Revised: October 13, 2017
Accepted: December 12, 2017
Published: January 11, 2018

## REFERENCES

Allen, G.F., Toth, R., James, J., and Ganley, I.G. (2013). Loss of iron triggers PINK1/Parkin-independent mitophagy. EMBO Rep. *14*, 1127–1135.

Ames, A., 3rd, Li, Y.Y., Heher, E.C., and Kimble, C.R. (1992). Energy metabolism of rabbit retina as related to function: high cost of Na+ transport. J. Neurosci. *12*, 840–853.

Antonucci, L., Fagman, J.B., Kim, J.Y., Todoric, J., Gukovsky, I., Mackey, M., Ellisman, M.H., and Karin, M. (2015). Basal autophagy maintains pancreatic acinar cell homeostasis and protein synthesis and prevents ER stress. Proc. Natl. Acad. Sci. USA *112*, E6166–E6174.

Beal, M.F., Brouillet, E., Jenkins, B.G., Ferrante, R.J., Kowall, N.W., Miller, J.M., Storey, E., Srivastava, R., Rosen, B.R., and Hyman, B.T. (1993). Neurochemical and histologic characterization of striatal excitotoxic lesions produced by the mitochondrial toxin 3-nitropropionic acid. J. Neurosci. *13*, 4181–4192.

Billia, F., Hauck, L., Konecny, F., Rao, V., Shen, J., and Mak, T.W. (2011). PTEN-inducible kinase 1 (PINK1)/Park6 is indispensable for normal heart function. Proc. Natl. Acad. Sci. USA *108*, 9572–9577.

Brouillet, E., Jacquard, C., Bizat, N., and Blum, D. (2005). 3-Nitropropionic acid: a mitochondrial toxin to uncover physiopathological mechanisms underlying striatal degeneration in Huntington's disease. J. Neurochem. *95*, 1521–1540.

Deas, E., Piipari, K., Machhada, A., Li, A., Gutierrez-del-Arroyo, A., Withers, D.J., Wood, N.W., and Abramov, A.Y. (2014). PINK1 deficiency in beta-cells increases basal insulin secretion and improves glucose tolerance in mice. Open Biol. *4*, 140051.

Doty, R.L. (2012a). Olfaction in Parkinson's disease and related disorders. Neurobiol. Dis. *46*, 527–552.

Doty, R.L. (2012b). Olfactory dysfunction in Parkinson disease. Nat. Rev. Neurol. *8*, 329–339.

Duran-Vilaregut, J., del Valle, J., Camins, A., Pallas, M., Pelegri, C., and Vilaplana, J. (2009). Blood-brain barrier disruption in the striatum of rats treated with 3-nitropropionic acid. Neurotoxicology *30*, 136–143.

Flinn, L.J., Keatinge, M., Bretaud, S., Mortiboys, H., Matsui, H., De Felice, E., Woodroof, H.I., Brown, L., McTighe, A., Soellner, R., et al. (2013). TigarB causes mitochondrial dysfunction and neuronal loss in PINK1 deficiency. Ann. Neurol. *74*, 837–847.

Gerasimenko, O.V., and Gerasimenko, J.V. (2012). Mitochondrial function and malfunction in the pathophysiology of pancreatitis. Pflugers Arch. *464*, 89–99.

Gong, G., Song, M., Csordas, G., Kelly, D.P., Matkovich, S.J., and Dorn, G.W., 2nd (2015). Parkin-mediated mitophagy directs perinatal cardiac metabolic maturation in mice. Science *350*, aad2459.

Grealish, S., Jonsson, M.E., Li, M., Kirik, D., Bjorklund, A., and Thompson, L.H. (2010). The A9 dopamine neuron component in grafts of ventral mesencephalon is an important determinant for recovery of motor function in a rat model of Parkinson's disease. Brain *133*, 482–495.

Huisman, E., Uylings, H.B., and Hoogland, P.V. (2004). A 100% increase of dopaminergic cells in the olfactory bulb may explain hyposmia in Parkinson's disease. Mov. Disord. *19*, 687–692.

Johnson, P.R., Dolman, N.J., Pope, M., Vaillant, C., Petersen, O.H., Tepikin, A.V., and Erdemli, G. (2003). Non-uniform distribution of mitochondria in pancreatic acinar cells. Cell Tissue Res. *313*, 37–45.

Kim, J., Byun, J.W., Choi, I., Kim, B., Jeong, H.K., Jou, I., and Joe, E. (2013). PINK1 deficiency enhances inflammatory cytokine release from acutely prepared brain slices. Exp. Neurobiol. *22*, 38–44.

Kluge, M.A., Fetterman, J.L., and Vita, J.A. (2013). Mitochondria and endothelial function. Circ. Res. *112*, 1171–1188.

Kondapalli, C., Kazlauskaite, A., Zhang, N., Woodroof, H.I., Campbell, D.G., Gourlay, R., Burchell, L., Walden, H., Macartney, T.J., Deak, M., et al. (2012). PINK1 is activated by mitochondrial membrane potential depolarization and stimulates Parkin E3 ligase activity by phosphorylating Serine 65. Open Biol. *2*, 120080.

Kumar, A., Tamjar, J., Waddell, A.D., Woodroof, H.I., Raimi, O.G., Shaw, A.M., Peggie, M., Muqit, M.M., and van Aalten, D.M. (2017). Structure of PINK1 and mechanisms of Parkinson's disease associated mutations. Elife *6*, https://doi.org/10.7554/eLife.29985.

Liang, C.L., Wang, T.T., Luby-Phelps, K., and German, D.C. (2007). Mitochondria mass is low in mouse substantia nigra dopamine neurons: implications for Parkinson's disease. Exp. Neurol. *203*, 370–380.

Markopoulou, K., Larsen, K.W., Wszolek, E.K., Denson, M.A., Lang, A.E., Pfeiffer, R.F., and Wszolek, Z.K. (1997). Olfactory dysfunction in familial parkinsonism. Neurology *49*, 1262–1267.

Martinez, D.P., and Freeman, W.J. (1984). Periglomerular cell action on mitral cells in olfactory bulb shown by current source density analysis. Brain Res. *308*, 223–233.

McWilliams, T.G., and Ganley, I.G. (2016). Life in lights: tracking mitochondrial delivery to lysosomes in vivo. Autophagy *12*, 2506–2507.

McWilliams, T.G., and Muqit, M.M. (2017). PINK1 and Parkin: emerging themes in mitochondrial homeostasis. Curr. Opin. Cell Biol. *45*, 83–91.

McWilliams, T.G., Prescott, A.R., Allen, G.F., Tamjar, J., Munson, M.J., Thomson, C., Muqit, M.M., and Ganley, I.G. (2016). mito-QC illuminates mitophagy and mitochondrial architecture in vivo. J. Cell Biol. *214*, 333–345.

Menzies, R.A., and Gold, P.H. (1971). The turnover of mitochondria in a variety of tissues of young adult and aged rats. J. Biol. Chem. *246*, 2425–2429.

Mizushima, N., Yamamoto, A., Matsui, M., Yoshimori, T., and Ohsumi, Y. (2004). In vivo analysis of autophagy in response to nutrient starvation using transgenic mice expressing a fluorescent autophagosome marker. Mol. Biol. Cell *15*, 1101–1111.

Mondelli, V., Vernon, A.C., Turkheimer, F., Dazzan, P., and Pariante, C.M. (2017). Brain microglia in psychiatric disorders. Lancet Psychiatry *4*, 563–572.

Narendra, D., Tanaka, A., Suen, D.F., and Youle, R.J. (2008). Parkin is recruited selectively to impaired mitochondria and promotes their autophagy. J. Cell Biol. *183*, 795–803.

Nishino, H., Shimano, Y., Kumazaki, M., and Sakurai, T. (1995). Chronically administered 3-nitropropionic acid induces striatal lesions attributed to dysfunction of the blood-brain barrier. Neurosci. Lett. *186*, 161–164.

Ordureau, A., Sarraf, S.A., Duda, D.M., Heo, J.M., Jedrychowski, M.P., Sviderskiy, V.O., Olszewski, J.L., Koerber, J.T., Xie, T., Beausoleil, S.A., et al. (2014). Quantitative proteomics reveal a feedforward mechanism for mitochondrial PARKIN translocation and ubiquitin chain synthesis. Mol. Cell *56*, 360–375.

Pacelli, C., Giguere, N., Bourque, M.J., Levesque, M., Slack, R.S., and Trudeau, L.E. (2015). Elevated mitochondrial bioenergetics and axonal arborization size are key contributors to the vulnerability of dopamine neurons. Curr. Biol. *25*, 2349–2360.

Park, M.K., Ashby, M.C., Erdemli, G., Petersen, O.H., and Tepikin, A.V. (2001). Perinuclear, perigranular and sub-plasmalemmal mitochondria have distinct functions in the regulation of cellular calcium transport. EMBO J. *20*, 1863–1874.

Perry, V.H., and Holmes, C. (2014). Microglial priming in neurodegenerative disease. Nat. Rev. Neurol. *10*, 217–224.

Petersen, O.H. (2012). Specific mitochondrial functions in separate subcellular domains of pancreatic acinar cells. Pflugers Arch. *464*, 77–87.

Pickrell, A.M., Huang, C.H., Kennedy, S.R., Ordureau, A., Sideris, D.P., Hoekstra, J.G., Harper, J.W., and Youle, R.J. (2015). Endogenous parkin preserves dopaminergic substantia nigral neurons following mitochondrial DNA mutagenic stress. Neuron *87*, 371–381.

Pickrell, A.M., and Youle, R.J. (2015). The roles of PINK1, parkin, and mitochondrial fidelity in Parkinson's disease. Neuron *85*, 257–273.

Poewe, W., Seppi, K., Tanner, C.M., Halliday, G.M., Brundin, P., Volkmann, J., Schrag, A.E., and Lang, A.E. (2017). Parkinson disease. Nat. Rev. Dis. Primers *3*, 17013.

Ponsen, M.M., Stoffers, D., Booij, J., van Eck-Smit, B.L., Wolters, E., and Berendse, H.W. (2004). Idiopathic hyposmia as a preclinical sign of Parkinson's disease. Ann. Neurol. *56*, 173–181.

Rojansky, R., Cha, M.Y., and Chan, D.C. (2016). Elimination of paternal mitochondria in mouse embryos occurs through autophagic degradation dependent on PARKIN and MUL1. Elife *5*, https://doi.org/10.7554/eLife.17896.

Sun, N., Yun, J., Liu, J., Malide, D., Liu, C., Rovira, I.I., Holmstrom, K.M., Fergusson, M.M., Yoo, Y.H., Combs, C.A., et al. (2015). Measuring in vivo mitophagy. Mol. Cell *60*, 685–696.

Sun, X., Liu, M., Hao, J., Li, D., Luo, Y., Wang, X., Yang, Y., Li, F., Shui, W., Chen, Q., et al. (2013). Parkin deficiency contributes to pancreatic tumorigenesis by inducing spindle multipolarity and misorientation. Cell Cycle *12*, 1133–1141.

Theiler, K. (1989). The House Mouse: Atlas of Embryonic Development (Springer-Verlag), p. 178.

Trifunovic, A., Wredenberg, A., Falkenberg, M., Spelbrink, J.N., Rovio, A.T., Bruder, C.E., Bohlooly, Y.M., Gidlof, S., Oldfors, A., Wibom, R., et al. (2004). Premature ageing in mice expressing defective mitochondrial DNA polymerase. Nature *429*, 417–423.

Uhlen, M., Fagerberg, L., Hallstrom, B.M., Lindskog, C., Oksvold, P., Mardinoglu, A., Sivertsson, A., Kampf, C., Sjostedt, E., Asplund, A., et al. (2015). Proteomics. Tissue-based map of the human proteome. Science *347*, 1260419.

Whitworth, A.J., and Pallanck, L.J. (2017). PINK1/Parkin mitophagy and neurodegeneration-what do we really know in vivo? Curr. Opin. Genet. Dev. *44*, 47–53.

Wong-Riley, M.T. (2010). Energy metabolism of the visual system. Eye Brain *2*, 99–116.

Yamano, K., and Youle, R.J. (2013). PINK1 is degraded through the N-end rule pathway. Autophagy *9*, 1758–1769.

Zhang, H., Bosch-Marce, M., Shimoda, L.A., Tan, Y.S., Baek, J.H., Wesley, J.B., Gonzalez, F.J., and Semenza, G.L. (2008). Mitochondrial autophagy is an HIF-1-dependent adaptive metabolic response to hypoxia. J. Biol. Chem. *283*, 10892–10903.

Zlokovic, B.V. (2011). Neurovascular pathways to neurodegeneration in Alzheimer's disease and other disorders. Nat. Rev. Neurosci. *12*, 723–738.

# STAR★METHODS

## KEY RESOURCES TABLE

| REAGENT or RESOURCE | SOURCE | IDENTIFIER |
|---|---|---|
| Antibodies | | |
| Sheep polyclonal anti-mouse Pten-induced putative kinase 1 (PINK1) (235–580aa) | MRC PPU Products & Reagents; This Study | S774C (DU17570) |
| Sheep polyclonal anti-mouse PINK1 (175–250 aa) | MRC PPU Products & Reagents; This Study | S086D (DU34559) |
| Sheep polyclonal anti-Mitochondrial Rho GTPase 1 (MIRO1) | MRC PPU Products & Reagents | S531D (DU40832) |
| Rabbit monoclonal anti-Vinculin | Abcam | Cat#: ab129002; RRID: AB_11144129 |
| Mouse monoclonal anti-Mitochondrial ATP Synthase Subunit Beta (ATPB) | Abcam | Cat#: ab14730; RRID: AB_301438 |
| Mouse monoclonal anti-mitochondrial dynamin like GTPase 1 (OPA1) | BD Biosciences | Cat#: 612606; RRID: AB_612606 |
| Rabbit polyclonal anti-Heat Shock Protein 60 (HSP60) | Cell Signalling Technology | Cat#: 4870S; RRID: AB_2295614 |
| Rabbit monoclonal anti-Pyruvate Dehydrogenase (PDH) | Cell Signalling Technology | Cat#: 3205S; RRID: AB_2162926 |
| Rabbit anti-Glial fibrillary acidic protein (GFAP) | DAKO | Cat#: Z0334; RRID: AB_10013382 |
| Rabbit polyclonal anti-Tyrosine Hydroxylase (TH) | Millipore | Cat#: AB152; RRID: AB_390204 |
| Sheep polyclonal anti-Tyrosine Hydroxylase (TH) | Millipore | Cat#: AB1542; RRID: AB_90755 |
| Mouse monoclonal anti-Tyrosine Hydroxylase (TH) [LNC1] | Millipore | Cat#: MAB318; RRID: AB_2201528 |
| Rabbit polyclonal anti-phospho-S65 Ubiquitin | Millipore | Cat#: ABS1513-I |
| Rabbit polyclonal anti-CDGSH Iron-Sulfur Domain-containing protein 1 (CISD1) | Proteintech | Cat#: 16006-1-AP; RRID: AB_2080268 |
| Mouse monoclonal anti-alpha Tubulin | Proteintech | Cat#: 66031-1-Ig RRID: AB_11042766 |
| Rat monoclonal anti-Haemagglutinin (HA)-Peroxidase | Roche | Cat#: 12013819001; RRID: AB_390917 |
| Rabbit polyclonal anti-Green Fluorescent Protein (GFP) | Roche (Sigma-Aldrich) | Cat#: 11814460001 RRID: AB_390913 |
| Rat monoclonal anti-lysosomal associated membrane protein 1 (LAMP1) (1D4B) | Santa Cruz Biotechnology | Cat#: sc-19992 RRID: AB_2134495 |
| Rabbit polyclonal anti-Translocase of Outer Membrane (Tom20) | Santa Cruz Biotechnology | Cat#: sc-11415; RRID: AB_2207533 |
| Rabbit polyclonal anti-Ionized calcium binding adaptor molecule 1 (Iba1) | Wako | Cat#: 019-19741; RRID: AB_839504 |
| Donkey Anti-Rabbit IgG H&L (Alexa Fluor 405) preadsorbed secondary antibody | Abcam | Cat#: ab175649; RRID: AB_2715515 |
| Goat anti-Rabbit IgG (H+L) Secondary Antibody, Pacific Blue | Life Technologies (Thermo Fisher Scientific) | Cat#: P-10994; RRID: AB_2539814 |
| Donkey anti-Sheep IgG (H+L) Cross-Adsorbed Secondary Antibody, Alexa Fluor 633 | Life Technologies (Thermo Fisher Scientific) | Cat#: A-21100; RRID: AB_2535754 |
| Goat anti-Mouse IgG (H+L) Highly Cross-Adsorbed Secondary Antibody, Alexa Fluor 647 | Life Technologies (Thermo Fisher Scientific) | Cat#: A-21236; RRID: AB_141725 |
| Goat anti-Rat IgG (H+L) Cross-Adsorbed Secondary Antibody, Alexa Fluor 633 | Life Technologies (Thermo Fisher Scientific) | Cat#: A-21094; RRID: AB_141553 |

(*Continued on next page*)

*Continued*

| REAGENT or RESOURCE | SOURCE | IDENTIFIER |
|---|---|---|
| Goat anti-Rabbit IgG (H+L) Secondary Antibody, HRP | Life Technologies (Thermo Fisher Scientific) | Cat#: 31460; RRID: AB_228341 |
| Goat anti-Rat IgG (H+L) Secondary Antibody, HRP | Life Technologies (Thermo Fisher Scientific) | Cat#: 31470; RRID: AB_228356 |
| Rabbit anti-Sheep IgG (H+L) Secondary Antibody, HRP | Life Technologies (Thermo Fisher Scientific) | Cat#: 31480; RRID: AB_228457 |
| Chemicals, Peptides, and Recombinant Proteins | | |
| Trypsin (MS Grade) | Thermo Fisher Scientific | Cat#: 90058 |
| 2-Chloroacetamide | Sigma-Aldrich | Cat#: C-0267 |
| Carbonyl cyanide 3-chlorophenylhydrazone (CCCP) | Sigma-Aldrich | Cat#: C2759 |
| 3-Nitropropionic acid (3-NPA) | Sigma-Aldrich | Cat#: N5636 |
| Deferiprone (DFP) | Sigma-Aldrich | Cat#: 379409 |
| VECTASHIELD Antifade Mounting Medium | Vector Laboratories | Cat#: H-1000; RRID: AB_2336789 |
| Recombinant TUBE Protein: His-Halo-UBA$^{UBQLN1}$ | MRC PPU Products & Reagents | Cat#: DU23799 |
| Experimental Models: Cell Lines | | |
| Human: HEK293 FT | Allen et al. (2013); This Study | N/A |
| Mouse: Primary & SV40 Immortalized Mouse Embryonic Fibroblasts | This study | N/A |
| Experimental Models: Organisms/Strains | | |
| Mouse model: *mito*-QC mice | McWilliams et al. (2016); This study (Mice generated by TaconicArtemis GmbH) | N/A |
| Mouse model: *Pink1* KO mice | Dr. L. Miguel Martins, Leicester UK; (Mice generated by Lexicon Pharmaceuticals, Inc.) | N/A |
| Mouse model: mCherry-GFP-*Map1lc3b* | This study (Mice generated by TaconicArtemis GmbH) | N/A |
| Recombinant DNA | | |
| Plasmid: pQCXIP puro HA-Parkin | MRC PPU Products & Reagents | DU55566 |
| GP2-293 Packaging Cell Line | Clontech | Cat#: 631458 |
| pVSV-G Vector | Clontech | Cat#: 631457 |
| Plasmid: pBabe Hygro SV40 DU40188 | MRC PPU Products & Reagents | DU40188 |
| Software and Algorithms | | |
| Volocity | PerkinElmer | http://cellularimaging.perkinelmer.com/downloads/ |
| Imaris | Bitplane | http://www.bitplane.com/imaris |
| Xcalibur v2.2$^{TM}$ | Thermo Scientific | https://www.thermofisher.com/order/catalog/product/OPTON-30487 |
| Mascot 2.4.1 | Matrix Science | http://www.matrixscience.com/ |
| Other | | |
| Zeiss LSM 710 Confocal Laser Scanning Microscope | Zeiss | N/A |
| Zeiss LSM 880 Confocal Laser Scanning Microscope with Airyscan | Zeiss | N/A |
| Thermo LTQ-Orbitrap Velos | Thermo Fisher Scientific | N/A |
| Thermo U3000 RSLC nano liquid chromatography system | Thermo Fisher Scientific | N/A |

## CONTACT FOR REAGENT AND RESOURCE SHARING

Further information and requests for resources and reagents should be directed to, and will be fulfilled by, the Lead Contact: Ian G. Ganley (i.ganley@dundee.ac.uk).

## EXPERIMENTAL MODEL AND SUBJECT DETAILS

### Animals

The *mito*-QC mouse model used in this study was generated as previously described (McWilliams et al., 2016) and crossed with *Pink1* heterozygote animals to produce WT and KO animals with endogenous *mito*-QC reporter. Autophagy reporter mice (*mCherry-GFP-Map1lc3b*, referred to as mCherry-GFP-LC3) were generated using targeted transgenesis by TaconicArtemis GmbH. Recombination-mediated cassette exchange (RMCE) was used to insert a CAG promoter cassette and the open reading frame for the mCherry-GFP-LC3 fusion protein including a Kozak sequence (GCCACC) into the mouse *Rosa26* locus (protein sequence of the mCherry-GFP-Map1lc3b fusion protein: MVSKGEEDNMAIIKEFMRFKVHMEGSVNGHEFEIEGEGEGRPYEGTQTAKLKVTKGGPLP FAWDILSPQFMYGSKAYVKHPADIPDYLKLSFPEGFKWERVMNFEDGGVVTVTQDSSLQDGEFIYKVKLRGTNFPSDGPVMQKKTMGW EASSERMYPEDGALKGEIKQRLKLKDGGHYDAEVKTTYKAKKPVQLPGAYNVNIKLDITSHNEDYTIVEQYERAEGRHSTGGMDELYKG GGSMVSKGEELFTGVVPILVELDGDVNGHKFSVSGEGEGDATYGKLTLKFICTTGKLPVPWPTLVTTLTYGVQCFSRYPDHMKQHDFFK SAMPEGYVQERTIFFKDDGNYKTRAEVKFEGDTLVNRIELKGIDFKEDGNILGHKLEYNYNSHNVYIMADKQKNGIKVNFKIRHNIEDGSV QLADHYQQNTPIGDGPVLLPDNHYLSTQSALSKDPNEKRDHMVLLEFVTAAGITLGMDELYKSGLGSMPSEKTFKQRRSFEQRVEDVR LIREQHPTKIPVIIERYKGEKQLPVLDKTKFLVPDHVNMSELIKIIRRRLQLNANQAFFLLVNGHSMVSVSTPISEVYESERDEDGFLYMVYA SQETFGTALAV, plus a hGH polyadenylation signal and additional polyadenylation signal). The RMCE vector was transfected into a TaconicArtemis C57BL/6 ES cell line containing RMCE docking sites in the *Rosa26* locus. Recombinant clones were isolated *via* positive–negative ($Neo^R$) selection. All mice in this study were maintained on a C57BL/6J background. Mice of both genders were used in all experiments, aged between 8-9.5 months old. Animals were subjected to the following husbandry conditions: mice were housed in temperature-controlled rooms at 21°C, with 45-65% relative humidity and 12h/12h light/dark cycle. Mice had *ad libitum* access to food and water, and were regularly subjected to health and welfare monitoring as standard (twice-daily). All mice in this study had automatic watering (0.2 micron sterile filtered), and were fed rodent diet "R&M No.3, 9.5 mm pelleted (irradiated)" consisting of (proximate analysis) 10% moisture, 4.16% crude oil, 21.86% crude protein, 4.33% crude fiber, 7.89 % Ash and 51.24% nitrogen-free extract; Special Diets Services, UK. All cages had corn-cob substrate (provided as a nest-pack) and sizzle-nest material provided. Environmental enrichment was provided for all animals, with a cardboard tunnel for amalgamated females, single-housed males and squabbling males. Cages were changed as needed, but all cages were changed on at least a two-weekly cycle. Genotyping was performed by diagnostic end-point PCR using genomic DNA isolated from tissue biopsy specimens. WT and KO alleles were detected using KOD Hot Start DNA polymerase (EMD Millipore) and manufacturer-recommended conditions. Genotyping for *mito*-QC was performed as previously described (McWilliams et al., 2016). For mCherry-GFP-LC3 autophagy reporter mice, genotyping was performed with the following primer sets, at an annealing temperature of 60°C: PRIMER 7336_5: CAAAGACC CCAACGAGAAGC; PRIMER 3579_152: hGH pA 3′ 1:CCAAGGCACACAAAAAACC; PRIMER 1114_1: CTCTTCCCTCGTGATCTGCA ACTCC; PRIMER 1114_2: CATGTCTTTAATCTACCTCGATGG; with expected amplicons of 299 bp (WT) and 614 (LC3 Reporter). All animal studies and breeding was approved by the University of Dundee ethical review committee, and further subjected to approved study plans by the Named Veterinary Surgeon and Compliance Officer (Dr. Ngaire Dennison) and performed under a UK Home Office project license in accordance with the Animal Scientific Procedures Act (ASPA, 1986).

### *In Vitro* Models

For experiments using primary MEFs, embryos were derived from time-mated pregnant females at E12-E16.5 and staged according to the criteria of (Theiler, 1989). Primary MEFs were generated from *mito*-QC *Pink1* embryos (sex unknown) using standard protocols, maintained in DMEM/20% FBS/penicillin-streptomycin at 37°C/5% $CO_2$ as previously described (McWilliams et al., 2016). For some experiments, we used immortalized MEFs (sex unknown, generated using standard procedures – stable transduction of SV40 Large T Antigen) maintained as above.

## METHOD DETAILS

### Stimulation of Mitophagy

Stimulations with CCCP (20 μM), 3-NPA (20 μM) and DFP (1 mM) were performed as previously described (Allen et al., 2013). For *in vivo* experiments, cohorts of adult *mito*-QC animals were administered I.P. injections of 3-NPA (Sigma Aldrich) for 24h and 7 days (50 mg/kg for 5 days, 75 mg/kg for two days), after which terminal anesthesia and trans-cardial perfusion were performed to obtain tissues for histological analyses.

### Histology, Immunochemistry & Confocal Microscopy

Immunocytochemistry, immunohistochemistry and microscopy were performed as previously described (McWilliams et al., 2016; Allen et al., 2013), with minor modifications. Following terminal anesthesia *via* I.P. administration of Euthatal, adult animals were trans-cardially perfused with PBS to remove excess blood. Tissues were rapidly harvested and processed by immersion fixation in freshly prepared 3.7% PFA at pH 7.0 in 0.2M HEPES. Transverse brain sections (200 μm) were acquired using a vibratome (Leica). For cryosectioning, tissues were cryoprotected in 30% (w/v) sucrose in PBS at 4°C. Coronal brain sections (40 μm) were acquired using sledge microtome equipped with a freezing stage (Leitz) and processed for free-floating immunohistochemistry. Non-neural organs were embedded in OCT (Sakura) and sectioned using a cryostat (Leica). The following primary antibodies were used: rat

anti-LAMP1 (Santa Cruz Biotechnology, Inc.); Rabbit, sheep and mouse anti-Tyrosine Hydroxylase (Millipore); Rabbit anti-Iba1 (Wako). Alexa-Fluor conjugated secondary antibodies were obtained from Life Technologies (Molecular Probes). Details of antibody catalog numbers/RRIDs are available in the associated Key Resources Table that accompanies this paper. VECTASHIELD Antifade Mounting Medium H-1000 was used to mount tissue sections on slides (Leica Surgipath). Images were acquired using a Zeiss LSM 710 Laser Scanning Microscope (Plan-Neofuar ×40 objective, NA 1.30; Plan Apochromat ×63 objective NA 1.4; Plan Apochromat ×20 objective, NA 0.8), or a Zeiss LSM880 Airyscan Confocal Scanning microscope (ZEISS; Plan Apochromat ×63 objective, NA 1.4) and processed using ZEISS Zen Software/Adobe Photoshop or Imaris (Bitplane) for 3D Isosurface Rendering. Images were digitally altered within linear parameters, with minimal adjustments to levels and linear contrast applied to all images.

### Tissue Harvesting and Preparation of Protein Lysates

Fresh brains were rapidly excised, placed in cold PBS and microdissected with ultrafine microknives under stereomicroscopy. Upon isolation, each brain region was collected in a single 1.5 ml microcentrifuge tube and immediately plunged into dry ice. Mouse extra-neural tissues (visceral tissues, skeletal muscle) were rapidly dissected, excised and rinsed briefly in ultra-chilled PBS to remove excess blood and snap frozen in liquid $N_2$. Tissue samples were stored at -80°C until ready for processing. To make protein extracts, all tissues (neural subregions and extra-neural tissues) were weighed and defrosted on wet ice in 5-fold mass excess of freshly prepared, ice-cold lysis buffer containing: 50 mM Tris/HCl, pH 7.5, 1 mM EDTA pH 8.0, 1 mM EGTA pH 8.0, 1% Triton X-100, 0.25 M sucrose, 150 mM NaCl, 2 mM sodium orthovanadate, 1 mM NaF, 10 mM Sodium glycerolphosphate, 1.15 mM Sodium Molybdate, 4 mM Sodium tartrate dehydrate, 100 mM 2-Chloroacetamide, 1 mM DTT and Complete protease inhibitor cocktail (Roche). All inhibitors and DTT were added immediately prior to use. Tissue homogenisation was performed using a probe sonicator at 4°C (Branson Instruments). Crude lysates were incubated at 4°C on wet ice for 30-45 min, before clarification by centrifugation at 14,000 *rpm* for 30 min at 4°C. Supernatants used for subsequent steps were carefully removed and either used for downstream biochemical analyses or snap-frozen and stored at −80°C.

### Immunoblotting

Briefly, 20 μg of denatured protein lysates were resolved by SDS-PAGE using precast 4–12% Novex Bis-Acrylamide gels (Thermo Fisher Scientific). Gels were electrotransfered to Immobilon-P PVDF membrane (Millipore) using a Bio-Rad Transblot Transfer Apparatus. Equal loading was evaluated by briefly incubating membranes with the reversible protein-binding dye, Ponceau-S (Sigma-Aldrich). Membranes were incubated with primary antibodies overnight at 4°C in either 5% (wt/vol) nonfat dry milk (Marvel) or 5% bovine serum albumin (BSA) diluted in TBST (Tris-buffered saline with 0.1% Tween-20). After washes in TBST, secondary detection was performed with HRP-conjugated secondary antibodies (1:5,000). Membranes were washed in TBST and developed using standard chemiluminescence with ECL (Amersham) and exposure to hyperfilm (GE Healthcare). MEF cells were processed for biochemical analyses as previously described (Allen et al., 2013). To detect ubiquitylated CISD1 and phospho ubiquitin in adult fibroblasts, ubiquitin capture was employed using recombinant TUBEs (His-Halo-UBA$^{UBQLN1}$ #: DU23799, PPAD, MRC PPU) as described in Ordureau et al. (2014).

### Detection of Endogenous PINK1 Protein in Mouse Tissues

To enrich and detect endogenous mouse PINK1 protein, the total pool was first immunoprecipitated by incubating with sheep anti-mouse PINK1 covalently-coupled to protein-G sepharose beads (1 μg per μl beads; #S774C, 3$^{rd}$ Bleed), from 0.3-5 mg of lysate. IPs were performed overnight (18 hours) at 4°C with constant agitation on a wheel shaker set to approximately 25 rpm and protected from light. Lysis buffer supplemented with inhibitors/DTT was used to wash beads. In total beads were subjected to two washes with NaCl 300 mM, and two with 150 mM NaCl. Protein was eluted in 2X LDS (NuPAGE), subjected to 95°C for 3 min and eluted using a SpinX column, after which reducing agent (beta-mercaptoethanol) was added. Samples were resolved by SDS-PAGE, as described above. Detection of endogenous PINK1 was performed by immunoblotting, using sheep anti-mouse PINK1 #S086D (1-2 μg/ml, 3$^{rd}$ Bleed) and developed using standard chemiluminescence. All antibodies to endogenous mouse PINK1 were generated by MRC PPU Reagents and Services, University of Dundee (http://mrcppureagents.dundee.ac.uk/).

### Detection of Endogenous Mouse PINK1 Protein in Brain Regions by Mass Spectrometry

Endogenous mouse PINK1 was immunoprecipitated as described above from sub-dissected adult brain regions (cortex, cerebellum) obtained from *Pink1* WT and KO mice. Lysates were resolved by SDS-PAGE and gels were processed for in-gel trypsin digestion. Briefly, bands were revealed using Colloidal Coomassie R-250 and a gel region corresponding to >50-75 kDa was excised under aseptic, keratin-free conditions. Following de-staining steps and reduction using 5mM DTT (65°C, 20 min with agitation) and alkylation by 20 mM chloroacetamide (RT, 20 min with agitation), samples were digested overnight at 30°C in mass spectrometry grade trypsin, in 50 mM Triethylamonium bicarbonate. Peptides were extracted with 100% acetonitrile (ACN), dried *via* Speed-Vac and samples were then submitted to the MRC PPU Mass Spectrometry Facility for further analysis. Briefly, peptides were analysed by liquid chromatography (LC)-MS/MS using a Thermo U3000 RSLC Nano Liquid Chromatography system (Thermo-Fisher Scientific) coupled to a Thermo LTQ-Orbitrap Velos mass spectrometer (Thermo-Fisher Scientific). To avoid any potential contamination from WT protein, *Pink1* KO samples were run initially, followed by a blank and then WT preparations. Data files were searched using Mascot (www.matrixscience.com). Extracted Ion chromatograms (XICs) were obtained from mass spectrometry RAW data files using Xcalibur v2.2 (Thermo-Fisher Scientific). The mass range used was the observed m/z value of the relevant peptides +/- 10 ppm.

## QUANTIFICATION AND STATISTICAL ANALYSIS

### Semi-Automated Quantitation

Images were processed with Volocity 6.3 Image Analysis Software (PerkinElmer) using algorithms developed to analyze object overlap and count individual structures. For all analyses, we obtained images using uniform random sampling by an experimenter blind to all conditions. All images in each experimental group were processed as a batch using identical protocols. All images were pre-filtered to suppress noise (3x3 median filter). The same strategy was used to quantify all non-immunolabelled *mito*-QC-labelled images using auto-thresholding to identify objects (mean intensity +3 standard deviations). Objects were filtered using a minimum size cutoff (0.16 $\mu m^2$) and touching objects were separated using a guide size (0.4 $\mu m^2$). Mitolysosomes were typically identified on the basis of thresholded red signal not overlapping with green (this condition was relaxed in the case of samples affected by heat-mediated dequenching of the *mito*-QC probe during tissue processing). Where dequenching or spectral-overlap was observed in some instances, we employed LAMP1-immunohistochemistry to verify the lysosomal nature of mitolysosomes and green channel/ combined with LAMP1 immunostaining was used as a proxy for mitolysosomes. In the case of immunolabels used to identify cells of interest in brain sections (TH, Iba1), we employed a autothresholding criterion of mean intensity +1.5 standard deviations. A minimum size of 0.16 $\mu m^2$ was required, and a "Fill Holes in Objects" processing step was applied. Blood vessels were identified on the basis of high green channel intensity and distinctive morphology. For fasting experiments, a fixed intensity threshold was established by autothresholding (mean +3 standard deviations) images and acquisition of a mean value from Control animals. This threshold was applied to both control and fasted animal images.

### Statistical Analysis

Data are depicted as scatter plots with each data point representing the mean value of an individual animal subject, ± standard error of the mean (SEM). Numbers of subjects are indicated in the respective figure legends. Statistical analyses were performed in GraphPad Prism and/or RStudio. Student's t-test was used for pairwise comparisons, whereas multiple comparisons were analyzed with one-way analysis of variance (ANOVA) and Bonferonni's *post hoc* test where indicated in the figure legends. No methods were used to determine whether the data met assumptions of the statistical approach.

**Cell Metabolism, Volume *27***

## Supplemental Information

# Basal Mitophagy Occurs Independently of PINK1 in Mouse Tissues of High Metabolic Demand

**Thomas G. McWilliams, Alan R. Prescott, Lambert Montava-Garriga, Graeme Ball, François Singh, Erica Barini, Miratul M.K. Muqit, Simon P. Brooks, and Ian G. Ganley**

## Basal mitophagy occurs independently of PINK1 in mouse tissues of high-metabolic demand

Thomas G. McWilliams, Alan R. Prescott, Lambert Montava-Garriga, Graeme Ball, François Singh, Erica Barini, Miratul M. K. Muqit, Simon P. Brooks and Ian G. Ganley

### Supplemental Figures

**Figure S1: Monitoring mitophagy/autophagy using *mito*-QC/mCherry-GFP-LC3 and mass spectrometry sequence coverage. Related to Figure 1.**

(A) DFP triggers mitophagy in *Pink1* WT and KO *mito*-QC MEFs. MEFs were treated with DMSO (Control) and DFP for 24 hours. Scale bar, 5 *μ*m. (B) Quantitation of mitophagy induced by iron-chelation in S1A (Two-way ANOVA, ****=$P$<0.0001). (C) Endogenous activation of the PINK1-Parkin pathway. Adult fibroblasts were established from *Pink1* WT and KO mice and stimulated ±CCCP for 18 hours. Enriched mitochondrial fractions were processed for ubiquitin capture by TUBE$^{UBA2}$ as previously described. Denatured protein extracts were subjected to SDS-PAGE and immunoblotting for the Parkin substrate, CISD1. Membranes were stripped and re-probed with antibodies to the PINK1 substrate, phospho-S65 Ubiquitin. (D) Targeting strategy for mCherry-GFP-LC3 mice used in the study, generated by Taconic Artemis GmbH. (E) Mouse PINK1 sequence coverage from WT cortex and cerebellum. Unique peptides highlighted in red exhibited a Mascot ion score >22 (indicating identity or extensive homology).

**Figure S2: *mito*-QC reveals mitophagy in the dopaminergic system *in vivo.* Related to Figure 2.**

(A) Immunoblot showing no differences in levels of GFP, and the OMM Protein Tom20 in total brain extracts from *Pink1* WT and KO *mito*-QC mice. (B) Isosurface volume render of midbrain dopaminergic neurons with high levels of mitochondrial turnover in cell bodies. Inset shows a representative image from the original raw stack used to generate the isosurface render. (C) Additional quantative parameters measured using *mito*-QC. No differences were observed between genotypes in the

mean size or shape of mitolysosomes in midbrain dopaminergic axons or OB PGNs (n.s.=*P*>0.05; *Student's t*-test). (D) Mitophagy in A16 DA periglomerular neurons of the olfactory bulb (OB); G denotes a glomerulus. Arrows indicate mitophagy within PGNs (E) Analyses of mitophagy in all PGN DA neurons from *Pink1* WT and KO animals reveal no differences between genotypes (Student's *t*-test; n.s.=*P*>0.05). All scale bars, 5 μm. (F) *mito*-QC illuminates the mitochondrial rich nature of olfactory glomeruli. Airysan imaging and isosurface rendering revealed an unexpected complex and dense mitochondrial meshwork in adult olfactory glomeruli, and the intimate associations with TH-positive A16 PGNs. (G) Maximum projection z-stack showing representative example of mitophagy in a GFAP-positive astrocyte *in vivo*. Scale bar, 5 *μ*m.

**Figure S3: *mito*-QC reveals mitophagy in highly metabolic tissues. Related to Figures 3 and 4.**

(A) Section of adult retina with associated extra-ocular muscle. Mitophagy is visible in the ONL, and in muscle shown in transverse and longitudinal orientations. (B) Mitophagy proceeds independently of the visual cycle. Representative images of ONL showing no differences in retinal mitophagy in animals during light or dark cycles. (C) Additional quantitative parameters of mitolysosomes and mitochondrial cell biology: no differences were observed (n.s.=*P*>0.05; *Student's t*-test), apart from a modest decrease in mitolysosome size in KO microglia (*=*P*<0.05; *Student's t*-test). (D) Airyscan image of adult pancreas from a *mito*-QC animal, showing distinct pools of mitochondria previously reported by Petersen and colleagues. PG-M: perigranular mitochondria; PN-M: perinuclear mitochondria; SP-M: subplasmalemmal mitochondria.

**Figure S4: Loss of PINK1 does not influence 3-NPA mitotoxicity *in vivo*. Related to Figure 4.**

(A) Analysis of mitophagy reveals no differences in the striata, striatal vasculature and hearts of *Pink1* WT and KO mice treated systemically with the brain-penetrant mitotoxin 3-NPA for 7 days (n.s.=*P*>0.05; One-Way ANOVA with *Bonferroni post-hoc* test). (B) 3-NPA does not activate the endogenous PINK1-Parkin pathway. IP shows stabilization of endogenous PINK1 protein with CCCP, but not 3-NPA treatment. (C) Composite tile-scan micrograph showing a transverse section of hindlimb skeletal

muscle. The highly oxidative fibers of the soleus (S) and highly glycolytic fibers of the white gastrocnemius (GC) are easily distinguished by mitochondrial content using GFP expression of *mito*-QC. Dashed lines delineate different anatomical boundaries of the GC-white (Gw), GC-mixed (Gm) and GC-red (Gr) zones. In this anatomical orientation, *mito*-QC reveals the lateral-medial gradient of glycolytic to oxidative muscle fibers. Shown are representative examples of GC/S muscles treated with 3-NPA from *Pink1* WT and KO *mito*-QC mice. Close-up images reveal the striking detail of the mitochondrial reticulum in different sub-regions. Scale bars = 500, 200 and 10 µm, respectively. (D) Quantitation of mitochondrial content as a function of GFP intensity. A non-significant, yet observable elevation is evident in the skeletal muscles of *Pink1* KO animals treated with 3-NPA. (E) Quantitation of mitophagy as a function of increasing mitochondrial content in 3-NPA treated *Pink1* WT and KO mice (*=$P<0.05$; 3-NPA treated *Pink1* WT vs. KO using One-Way ANOVA, and *Bonferroni*'s post-hoc test to compare all conditions within regions).

# Figure S1

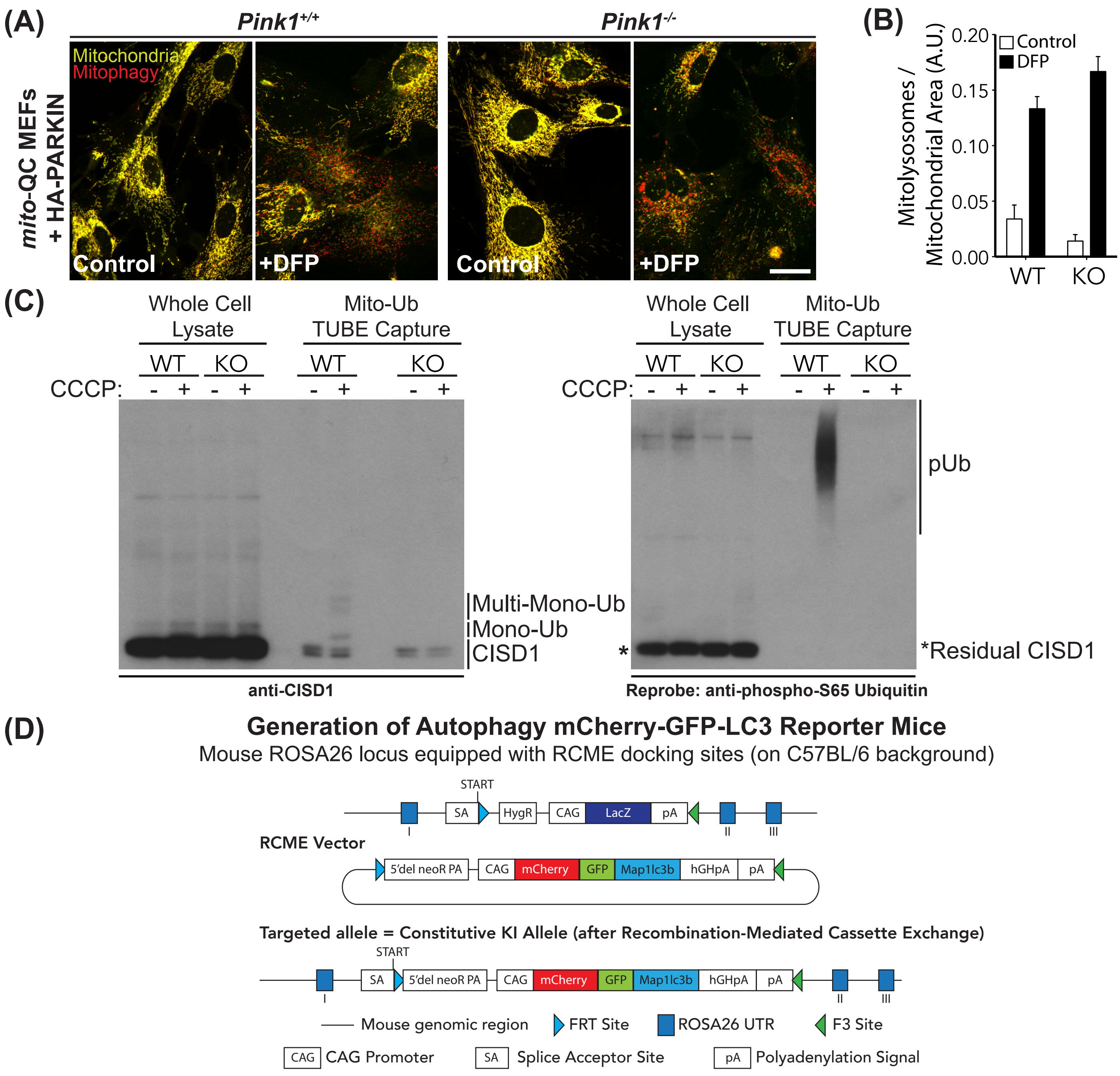


(E)

PINK1 peptides identified from PINK1 IP by mass spectrometry

| Cortex (CTX) WT - 21% Sequence Coverage | Cerebellum (CB) WT - 18% Sequence Coverage |
|---|---|
| 1 MAVRQALGRG LQLGRALLLR FAPKPGPLFG WGK**PGPAAAW** **GRGERPGQVV** | 1 MAVRQALGRG LQLGRALLLR FAPK**PGPLFG** **WGKPGPAAAW** **GRGERPGQVV** |
| 51 **SPGAQPR**PVG LPLPDRYRFF R**QSVAGLAAR** IQRQFMVRAR GGAGPCGRAV | 51 **SPGAQPR**PVG LPLPDRYRFF R**QSVAGLAAR** IQRQFMVRAR GGAGPCGRAV |
| 101 FLAFGLGLGL IEEKQAEGRR **AASACQEIQA** **IFTQK**TKRVS DPLDTRCWQG | 101 FLAFGLGLGL IEEKQAEGRR **AASACQEIQA** **IFTQK**TKRVS DPLDTRCWQG |
| 151 FR**LEDYLIGQ** **AIGK**GCNAAV YEATMPTLPQ HLEKAKHLGL IGKGPDVVLK | 151 FR**LEDYLIGQ** **AIGK**GCNAAV YEATMPTLPQ HLEKAKHLGL IGKGPDVVLK |
| 201 **GADGEQAPGT** **PTFPFAIK**MM WNISAGSSSE AILSK**MSQEL** **VPASRVALAG** | 201 **GADGEQAPGT** **PTFPFAIK**MM WNISAGSSSE AILSK**MSQEL** **VPASR**VALAG |
| 251 **EYGAVTYR**RS RDGPKQLAPH PNIIRVFRAF TSSVPLLPGA LADYPDMLPP | 251 EYGAVTYRRS RDGPKQLAPH PNIIRVFRAF TSSVPLLPGA LADYPDMLPP |
| 301 HYYPEGLGHG RTLFLVMKNY PCTLR**QYLEE** **QTPSSR**LATM MTLQLLEGVD | 301 HYYPEGLGHG RTLFLVMKNY PCTLR**QYLEE** **QTPSSR**LATM MTLQLLEGVD |
| 351 HLVQQGIAHR DLKSDNILVE WDSDGCPWLV ISDFGCCLAD QHVGLR**LPFN** | 351 HLVQQGIAHR DLKSDNILVE WDSDGCPWLV ISDFGCCLAD QHVGLRLPFN |
| 401 **SSSVER**GGNG SLMAPEVSTA HSGPSAVIDY SKADTWAVGA IAYEIFGLAN | 401 SSSVERGGNG SLMAPEVSTA HSGPSAVIDY SKADTWAVGA IAYEIFGLAN |
| 451 PFYGQGSAHL ESRSYQEAQL PEMPESVPPE ARRLVRSLLQ REASKRPSAR | 451 PFYGQGSAHL ESRSYQEAQL PEMPESVPPE ARRLVRSLLQ REASKRPSAR |
| 501 LAANVLHLSL WGEHLLALKN LKLDKMIAWL LQQSAATLLA DRLREKSCVE | 501 LAANVLHLSL WGEHLLALKN LKLDKMIAWL LQQSAATLLA DRLREKSCVE |
| 551 TKLQMLFLAN LECEALCQAA LLLSSWRAAP | 551 TKLQMLFLAN LECEALCQAA LLLSSWRAAP |

# Figure S2

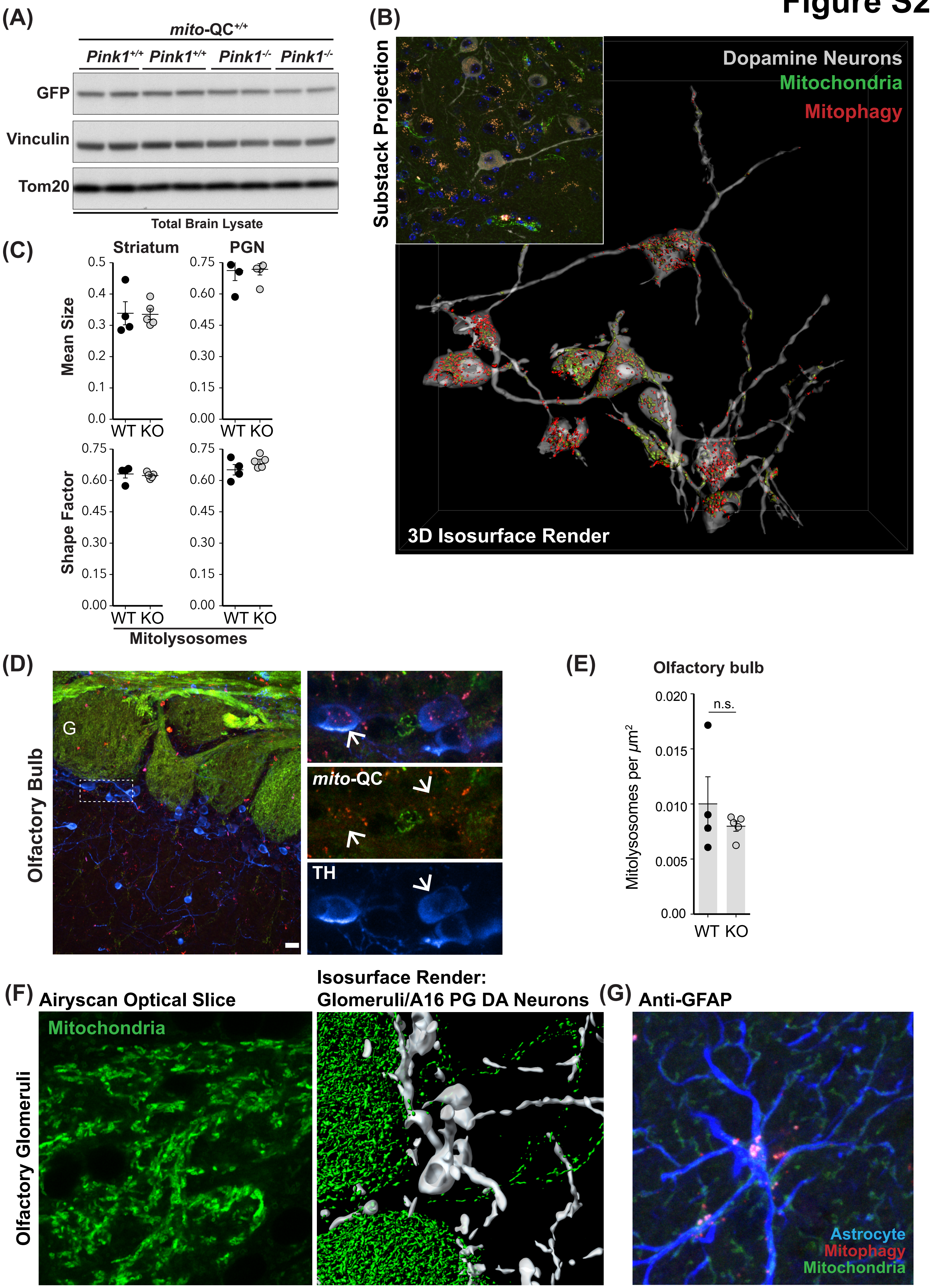

(A)
mito-QC+/+
Pinkl+/+ Pink1+/+ Pink1-/- Pink1-/-
GFP
Vinculin
Tom20
Total Brain Lysate
(B)
Substack Projection
Dopamine Neurons
Mitochondria
Mitophagy
3D Isosurface Render
(C)
Striatum
PGN
Mean Size
Shape Factor
WT KO
Mitolysosomes
(D)
Olfactory Bulb
G
mito-QC
TH
(E)
Olfactory bulb
n.s.
Mitolysosomes per μm²
WT KO
(F) Airyscan Optical Slice
Mitochondria
Olfactory Glomeruli
Isosurface Render:
Glomeruli/A16 PG DA Neurons
(G) Anti-GFAP
Astrocyte
Mitophagy
Mitochondria

# Figure S3

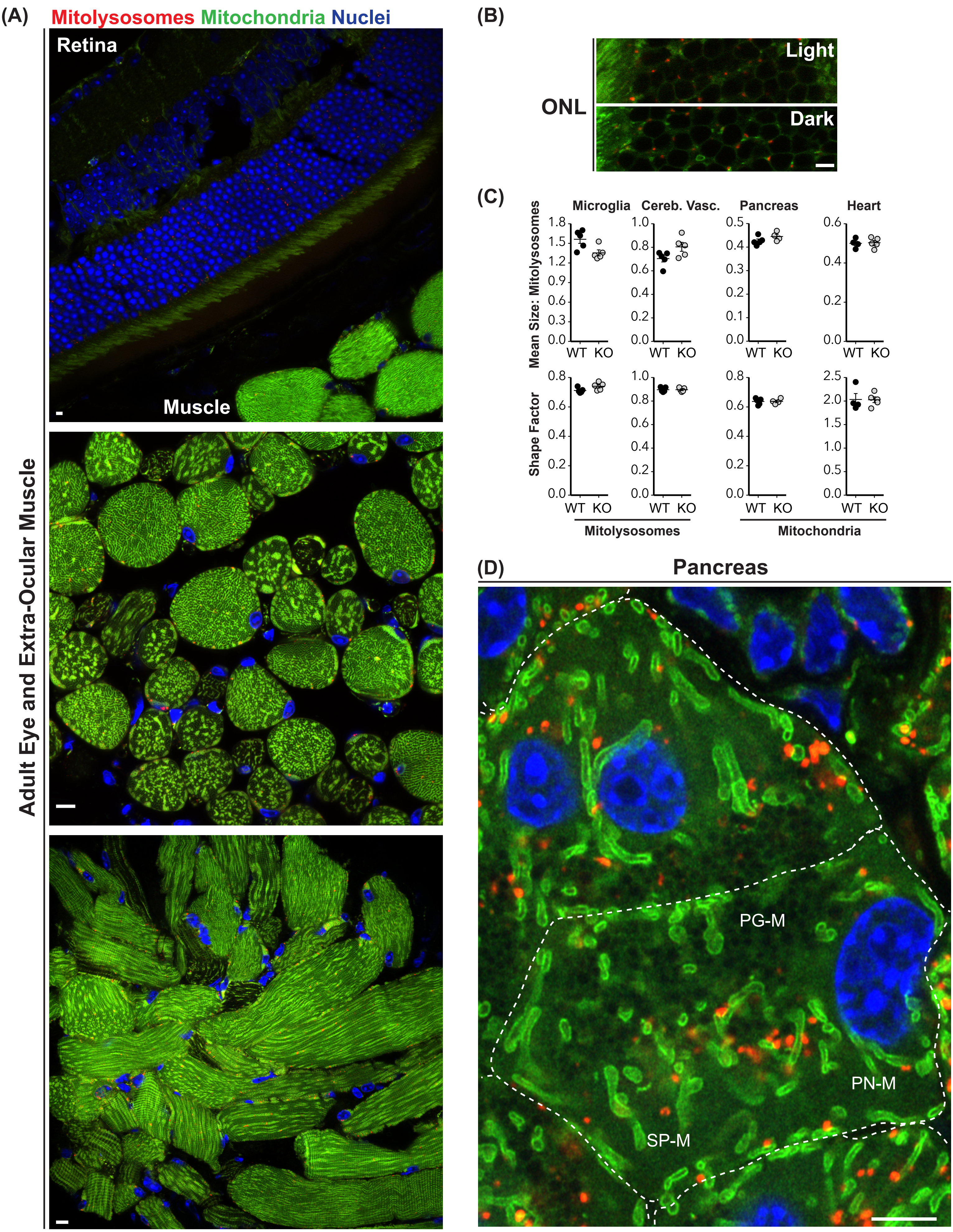

(A)
Mitolysosomes Mitochondria Nuclei
Retina
Muscle
Adult Eye and Extra-Ocular Muscle
(B)
ONL
Light
Dark
(C)
Mean Size: Mitolysosomes
Microglia
Cereb. Vasc.
Pancreas
Heart
Shape Factor
WT KO
Mitolysosomes
Mitochondria
(D)
Pancreas
PG-M
PN-M
SP-M

# Figure S4

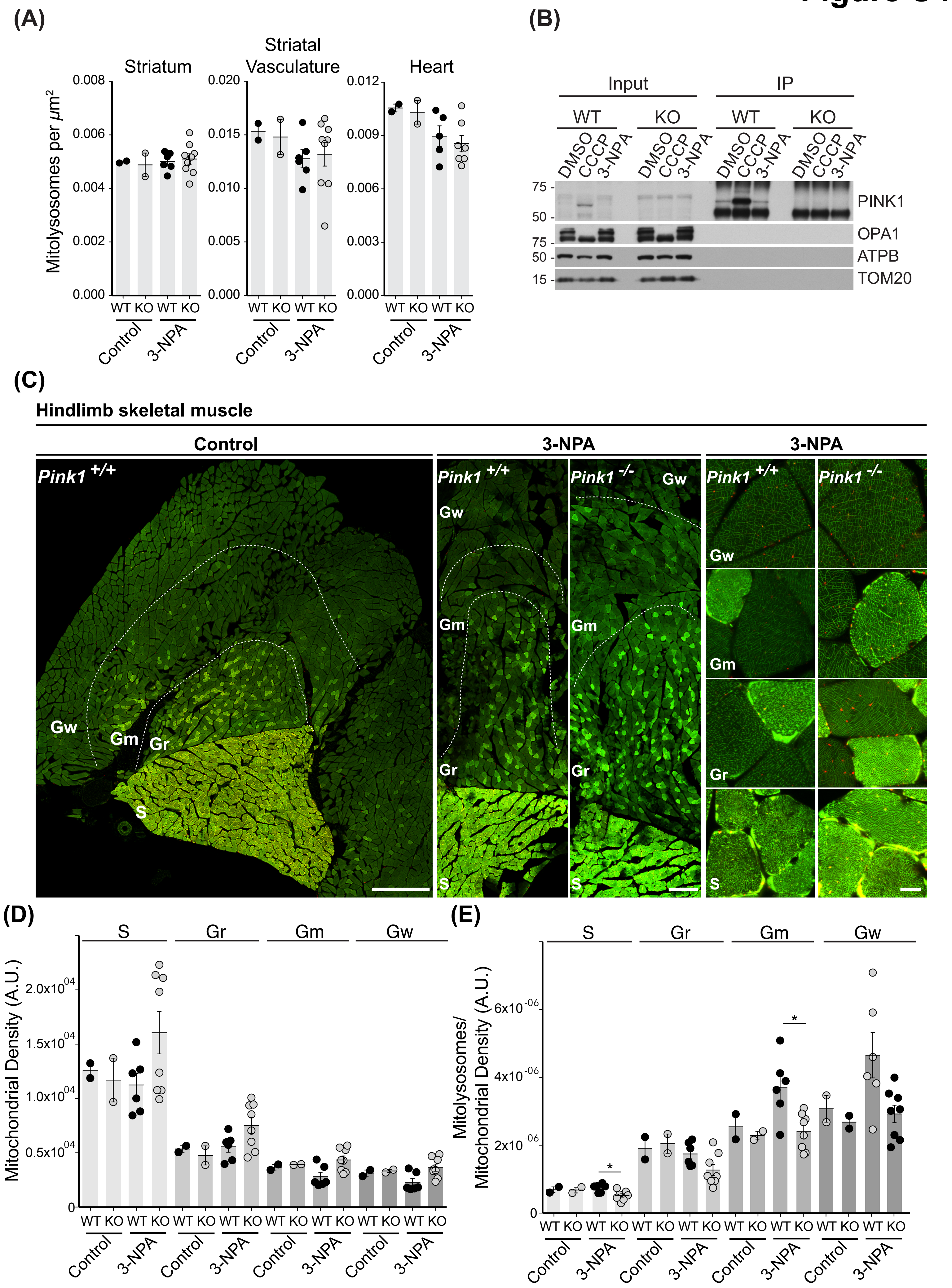

(A)
Striatum
Striatal Vasculature
Heart
Mitolysosomes per μm²
WT KO WT KO
Control
3-NPA
(B)
Input
IP
WT
KO
DMSO CCCP 3-NPA
PINK1
OPA1
ATPB
TOM20
(C)
Hindlimb skeletal muscle
Control
3-NPA
Pink1 +/+
Pink1 -/-
Gw
Gm
Gr
S
(D)
Mitochondrial Density (A.U.)
(E)
Mitolysosomes/ Mitochondrial Density (A.U.)
